# The Influence of Halide Substituents on the Structural and Magnetic Properties of Fe_6_Dy_3_ Rings

**DOI:** 10.3389/fchem.2020.00701

**Published:** 2020-08-14

**Authors:** Irina A. Kühne, Christopher E. Anson, Annie K. Powell

**Affiliations:** ^1^Institut für Anorganische Chemie, KIT (Karlsruhe Institute of Technology), Karlsruhe, Germany; ^2^School of Physics, University College Dublin (UCD), Dublin, Ireland; ^3^Institut für Nanotechnologie, KIT (Karlsruhe Institute of Technology), Eggenstein-Leopoldshafen, Germany

**Keywords:** single molecule magnet (SMM), iron, dysprosium, cyclic coordination cluster, substituent effect

## Abstract

We report the synthesis and magnetic properties of three new nine-membered Fe(III)-Dy(III) cyclic coordination clusters (CCCs), with a core motif of [Fe_6_Dy_3_(μ-OMe)_9_(vanox)_6_(X-benz)_6_] where the benzoate ligands are substituted in the para-position with X = F (**1**), Cl (**2**), Br (**3**). Single crystal X-ray diffraction structure analyses show that for the smaller fluorine or chlorine substituents the resulting structures exhibit an isostructural Fe_6_Dy_3_ core, whilst the 4-bromobenzoate ligand leads to structural distortions which affect the dynamic magnetic behavior. The magnetic susceptibility and magnetization of **1**-**3** were investigated and show similar behavior in the dc (direct current) magnetic data. Additional ac (alternating current) magnetic measurements show that all compounds exhibit frequency-dependent and temperature-dependent signals in the in-phase and out-of-phase component of the susceptibility and can therefore be described as field-induced SMMs. The fluoro-substituted benzoate cluster **1** shows a magnetic behavior closely similar to that of the corresponding unsubstituted Fe_6_Dy_3_ cluster, with U_eff_ = 21.3 K within the Orbach process. By increasing the size of the substituent toward 4-chlorobenzoate within **2**, an increase of the energy barrier to U_eff_ = 36.1 K was observed. While the energy barrier becomes higher from **1** to **2**, highlighting that the introduction of different substituents on the benzoate ligand in the *para*-position has an impact on the magnetic properties, cluster **3** shows a significantly different SMM behavior where U_eff_ is reduced in the Orbach regime to only 4.9 K.

## Introduction

Cyclic coordination clusters (CCCs) consisting of paramagnetic ions are of interest since these can show unusual physical and chemical properties arising from the cyclisation of a short chain of metal ions to give a finite molecular species (Larsen et al., 2003; Cador et al., 2004, 2005; Tang et al., 2006; Timco et al., 2009; Whitehead et al., 2013; Ungur et al., 2014; Ferrando-Soria et al., 2015; Gysler et al., 2016; Langley et al., 2019). For 4f containing cycles this includes stabilization of toroidal moments (Waldmann, 2005; Chibotaru et al., 2008; Ungur et al., 2012), whereas for 3d heterometallic cycles it has been shown that these can function as qubits and even be incorporated into logic gates (Troiani et al., 2005). Cyclic 3d/4f clusters where 3d = Fe(III) offer new opportunities in chiral separation (Baniodeh et al., 2013), magnetic resonance imaging (MRI) (Guthausen et al., 2015; Ranzinger et al., 2016) and the magnetocaloric effect (Botezat et al., 2017; Schmidt et al., 2017b). The Fe/4f cyclic coordination clusters show perhaps the most exotic behavior and can be produced in different nuclearities, such as [Fe_2_Ln_2_] (Song et al., 2013; Pugh et al., 2016; Alexandru et al., 2018), [Fe_3_Ln_2_] (Baniodeh et al., 2013), [Fe_4_Dy_4_] (Schray et al., 2010; Chen et al., 2015), [Fe_4_Ln_2_] (Schmidt et al., 2012, 2017a; Baniodeh et al., 2013; Botezat et al., 2017, 2019b; Chen et al., 2017), [Fe_5_Ln_3_] (Baniodeh et al., 2013), [Fe_6_Ln_3_] (Kühne et al., 2016; Botezat et al., 2019a), [Fe_6_Ln_4_] (Botezat et al., 2019b), [Fe_8_Ln_8_] (Zhang et al., 2020), [Fe_10_Ln_10_] (Baniodeh et al., 2013, 2014), [Fe_16_Ln_4_] (Baniodeh et al., 2011), and [Fe_18_Ln_6_] (Botezat et al., 2017). The Fe_10_Gd_10_ system demonstrated the potential of cyclized systems to show solid state properties on a molecular length scale—in this case, a quantum critical point tipping the system toward a ferromagnetic ultra-high spin state of S = 60 with a choice of at least 10,000 ground state configurations, making this a system with polynary rather than binary prospects (Baniodeh et al., 2018). A particularly fascinating aspect to these systems is provided by examples where the components of the chain are not simply alternating iron and 4f ions (Schmidt et al., 2012, 2017a; Baniodeh et al., 2013; Botezat et al., 2017, 2019b). This behavior was recently reported for the family of Fe_6_Ln_3_ clusters (Kühne et al., 2016) where the repeating unit can be regarded as {Fe_2_Ln}_3_ and three of these give an {Fe_6_Ln_3_} cycle. The repeating units within big clusters can vary, but it is also possible to have a situation where there is no true cyclisation of a fundamental building block. This can be seen for example in the arrangement within [Fe_4_Ln_2_] clusters where some structures can be described in terms of a {FeLn_2_}_2_ cycle as shown by Schmidt et al. (2012), Botezat et al. (2019b), and Schmidt et al. (2017a) which is very different from the cyclized strand of {FeLn_4_Fe} described in Baniodeh et al. (2013). Other repeating units, such as {Fe_3_Dy} have been reported in the largest example of Fe/4f coordination cluster where the basic unit is repeated six times to give an {Fe_18_Dy_6_} cycle (Botezat et al., 2017).

Odd-numbered rings are rare, with only a few examples (Baniodeh et al., 2013; Kühne et al., 2016; Botezat et al., 2019a), such as provided by our non-anuclear Fe_6_Ln_3_ clusters (Kühne et al., 2016), but they hold promise for discovering new exotic magnetic phenomena including single molecule magnetism, magnetocaloric effect, MRI contrast reagents, and toroidal arrangements of anisotropic 4f spin centers. It has previously been shown that electron-withdrawing/donating substituents on the phenyl ring of benzoic acids can lead to small changes in the structural environment and therefore have an impact on the magnetic properties of Single Molecule Magnets (SMMs) (Mereacre et al., 2011; Habib et al., 2013). Even small variations in the ligand field of lanthanide ions can have a significant influence on the slow relaxation process responsible for SMM behavior (Peng et al., 2016).

**Scheme 1:**
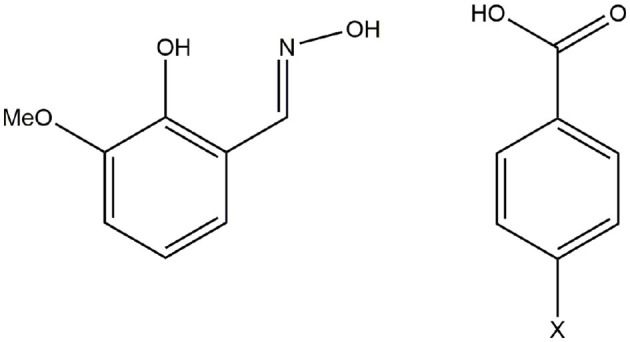
H_2_vanox (left) and 4-parasubstituted benzoic acid (right).

Here, we investigate the influence of para-substituents of the coordinated benzoate ligand on the structural and magnetic properties of [Fe_6_Dy_3_] cyclic coordination clusters. This systematic study led to three new crystal structures of cyclic [Fe_6_Dy_3_] clusters, with a core motif of [Fe_6_Dy_3_(μ-OMe)_9_(vanox)_6_(X-benz)_6_] where vanox^−2^ is doubly-deprotonated *o*-vanillinoxime, and the benzoate ligands are para-substituted with X = F (**1**), Cl (**2**), Br (**3**) (Scheme 1). The subtle changes in the non-coordinating part of the ligand, give insights into the structure-directing properties of the ligand as well as the magnetic properties of the clusters. The change of the coordination preferences of the bridging benzoate with *para-*substituent variation was monitored by magnetic susceptibility measurements. We compare the static and dynamic susceptibility data of the *para-*substituted **1**-**3** and the unsubstituted cyclic coordination clusters to determine the role of the electron-withdrawing group.

## Results

### Synthetic Route

The synthesis of **1**-**3** was achieved in a one pot reaction; for full synthetic details see section Materials and Methods. A ligand solution containing *o*-vanillinoxime, H_2_vanox, and sodium methoxide, NaOMe, dissolved in methanol was added to a solution of FeCl_2_·4H_2_O and Dy(NO_3_)_3_·6H_2_O in methanol with the respective *para*-halide-substituted benzoic acid as depicted in Scheme 1. Small adjustments in terms of solvents and amount of base to allow for the different pK_a_ of the *para*-substituted benzoic acids were made as outlined in section Materials and Methods. In this way, dark red single crystals of three nine-membered [Fe_6_Dy_3_(μ-OMe)_9_(vanox)_6_(X-benz)_6_] cyclic coordination clusters **1**-**3** were obtained for the 4-fluorobenzoate, [Fe_6_Dy_3_(μ-OMe)_9_(vanox)_6_(F-benz)_6_]^.^12MeOH^.^H_2_O (**1**), the 4-chlorobenzoate, [Fe_6_Dy_3_(μ-OMe)_9_(vanox)_6_(Cl-benz)_6_]^.^13MeOH (**2**), and the 4-bromobenzoate, [Fe_6_Dy_3_(μ-OMe)_9_(vanox)_6_(Br-benz)_6_(MeOH)]^.^ 9MeOH (**3**).

### Structural Details

**Figure 1:**
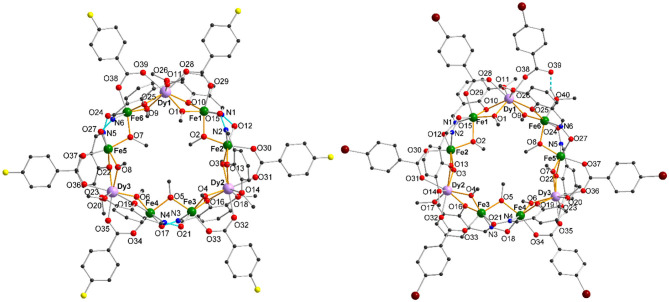
Molecular structure of [Fe_6_Dy_3_(μ-OMe)_9_(vanox)_6_(F-benz)_6_] cyclic coordination clusters (**1**) (left) and of [Fe_6_Dy_3_(μ-OMe)_9_(vanox)_6_(Br-benz)_6_(MeOH)] (**3**) (right) with H-bonds highlighted as turquoise dashed lines, (solvents and H atoms omitted for clarity).

**1**-**3** crystallize in the triclinic space group **P1̄**, where **1** and **2** crystallize isomorphically with 13 solvent molecules found in the crystal lattice. For **1**, there are 12 methanol molecules and one water molecule and for **2**, there are 13 methanol molecules. In contrast, **3** crystallizes with 9 molecules of methanol in the unit cell and incorporates a tenth methanol within an iron coordination sphere of the cluster as outlined below. A table summarizing the crystallographic details can be found in section Materials and Methods. The structure of the coordination cluster within **1** is taken as a representative of the isomorphous structures **1** and **2**. The structures of **1** and **3** are shown in Figure 1. The nonanuclear metal core of **1**-**3** consists of three Fe(III) dimeric units which are linked together through single Dy(III) ions to form a ring and this core motif has been previously reported for the Dy(III) and other Ln(III) compounds where the unsubstituted benzoate was used as a bridging co-ligand (Kühne et al., 2016).

**1** and **2** are analogous to the previously reported structure, with the six substituted benzoates in a 1,1′-μ-bridging mode, linking together each end of the dinuclear iron units to the intervening Dy(III) centers. Within **3** one of the Br-benzoates does not bridge between the Fe(III) and Dy(III) ions anymore, but coordinates only to the Dy(III) ion (Figure 1). The vacant coordination site on the Fe(III) center, Fe(6), is filled by O(40) of the methanol ligand. The hydrogen of this methanol forms a hydrogen-bond to the non-coordinated Br-benzoate O(39), which is highlighted as a turquoise dashed bond in Figure 1.

**Figure 2:**
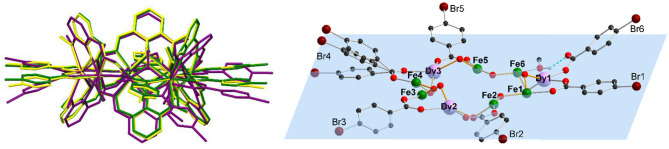
Structure overlap of **1**-**3** by superposition of all nine metal centers (left) (**1**: 4-fluorobenzoate (yellow), **2**: 4-chlorobenzoate (green) and **3**: 4-bromobenzoate (purple)), and plane through all nine metal centers (blue) of **3**, [Fe_6_Dy_3_(μ-OMe)_9_(vanox)_6_(Br-benz)_6_(MeOH)], (right), highlighting the deviation of the benzoate rings tilted out of the plane (all other ligands omitted for clarity, except for the 4-bromobenzoates and bridging methoxy groups).

Even though **1** and **2** crystallize isomorphously and show similar bond lengths and angles, the differences become more pronounced by overlapping the structures in Figure 2, where **1**-**3** are shown as capped stick models with **1** in yellow, **2** in green, and **3** in purple. The metal centers of all clusters almost overlap, highlighting the similarity of the M^...^M distances (Table S1) within **1**-**3** (Figure S1). While the vanox^2−^ and the μ_2_-bridging methoxy groups of **1** and **2** do overlap, there are some differences visible, especially for the coordinating benzoate ligands, which become more pronounced in the side view (Figure 2). By increasing the halide radius from F via Cl to Br, it becomes clear, that the benzoate ligands bend out of the metal plane, and this bending seems to be too much in case of **3**, which then leads to the break in connectivity observed in this structure. Additionally, the steric demand of the intramolecular H-bond of the unidentate bromobenzoate forces the latter out of the metal plane, which is defined by all nine Fe(III) and Dy(III) centers (Figure 2). The tilting angles of the benzoate ligands within **1**-**3** are summarized in Table S2, with small angles in the range 2–20° for the isomorphous clusters **1** and **2**, while **3** exhibits stronger tilting of 9.4–34.2° for the μ_2_-coordinated benzoates. The monodentate bromobenzoate Br6, exhibits the largest tilting out of the metal plane with an angle of 38.9°.

In order to see the differences between the structures which can lead to the tuning of the magnetic behavior, the bond lengths around the Fe(III) centers are summarized in Table S3. Table S4 summarizes all Dy-O bond lengths, and both sets of data are compared to the unsubstituted Fe_6_Dy_3_ cluster. All Fe(III) centers are hexa-coordinate with five oxygen atoms and one nitrogen atom in the coordination sphere. The Fe-O bond lengths within **1**-**3** are 1.93–2.08 Å and the Fe-N bond lengths are 2.09-2.13 Å, similar to those for the unsubstituted cluster. Although, **3** shows the disorder of one benzoate ligand, where the carboxylate oxygen of one of the Fe(III) centers, Fe6, is replaced by a coordinating methanol, this Fe-O bond length is in the same range as the respective Fe-O_carboxylate_ bonds within this cluster as well as compared to **1** and **2**.

Taken together, the Dy-O bond lengths are in a rather wide range, 2.23–2.56 Å. But if they are classified according to the nature of the coordinated oxygen, they now form four non-overlapping ranges, and Table 1 shows the ranges and mean values for the different Dy-O types in **1**-**3** and the unsubstituted Fe_6_Dy_3_ cluster. These clearly highlight the relative hardness of the different oxygen species coordinated to the Dy(III) centers, with the μ_2_-bridging methoxides exhibiting the shortest Dy-O distances, followed by those involving oxygens from the bridging carboxylates, the bridging phenoxides, while the neutral vanox methoxy groups unsurprisingly form the longest Dy-O bonds. The corresponding bond length for the monodentate bromobenzoate in **3** (Dy1-O38: 2.234 Å) was not included within the data for the bridging carboxylates in Table 1 as it is much shorter, and in fact it falls toward the lower end of the methoxide Dy-O range. This indicates that the “unplugging” of that benzoate from the Fe(III) center has increased the negative charge and hardness of O38, and this may have a significant effect on the electrostatic field around Dy1 in **3**.

**Table 1:** Dy-O bond length ranges and averages (in Å) in **1-3** and the unsubstituted cluster (Kühne et al., 2016).

**Dy-O (Å)**	**Range**	**Average**
Dy-O (μ-OMe)	2.228–2.282	2.259
Dy-O (μ-carboxylate)	2.282–2.352	2.316
Dy-O (μ-phenoxo)	2.361–2.431	2.392
Dy-O (vanox-OMe)	2.472–2.577	2.518

### Angular Distortion Analysis of Fe(III) Centers

As previously shown in [Fe_6_Ln_3_(μ-OMe)_9_(vanox)_6_(benz)_6_] (Kühne et al., 2016), the magnetic behavior of the clusters is dominated by the three dimeric Fe(III) units within the cluster which are antiferromagnetically coupled and the strength of the coupling can be influenced by the bridging angle between the Fe(III) centers as well as the distortion from the octahedral environment (Gorun and Lippard, 1991; Werner et al., 2001). The angular distortion from the octahedral environment around the Fe(III) centers also has an impact on the strength of the magnetic coupling. Therefore the overall distortion of the Fe(III) centers can be described by the angular distortion Σ and the torsional distortion Θ which are defined by formulas (1) and (2) by McKee (Drew et al., 1995), which is a common procedure to describe spin crossover compounds (Marchivie et al., 2005; Halcrow et al., 2019; Kühne et al., 2020).

(1) $$\begin{array}{l} \Sigma =\sum_{i=1}^{12}|90-\phi_{i}| \end{array}$$

(2) $$\begin{array}{l} \Theta =\sum_{j=1}^{24}|60-\theta_{j}| \end{array}$$

The degree of trigonal distortion can be described on the one hand by Σ, which shows the local angular deviation from the octahedral angle of 90° and, on the other hand, by Θ which is the sum of the deviation of the triangular faces from 60°, measured by the torsion angle between two adjacent coordinated oxygen atoms of the two triangles. Σ describes the sum of all 12 cis octahedral angles which should be ideally 90° and Θ describes the sum of all 24 unique torsion angles which define the degree of twist from octahedral toward trigonal prismatic geometry. The twist angle, θ, is defined between opposite triangular faces where θ = 0° for a trigonal prism and θ = 60° for an octahedron, leading to Θ = 1440.0° for a trigonal prism. For an octahedron, both Σ and Θ would be 0. Both Σ and Θ have been calculated for **1**-**3** as well as for the unsubstituted Fe_6_Dy_3_ cluster, using OctaDist 2.6.1 (Ketkaew et al., 2019) and the results are summarized in Table 2.

**Table 2:** Calculated Σ (top) and Θ (bottom) values (in °), together with the average values within one cluster and the average deviation per angle using OctaDist 2.6.1 (Ketkaew et al., 2019) for the hexacoordinated Fe(III) ions within 1–3 in comparison to the unsubstituted Fe_6_Dy_3_ cluster.

**Cluster**	**Fe1**	**Fe2**	**Fe3**	**Fe4**	**Fe5**	**Fe6**	**Σ_average_**	**av/angle**
[Fe_6_Dy_3_(benz)_6_]	87.41	80.34	92.77	87.41	80.34	92.77	86.84	7.24
[Fe_6_Dy_3_(F-benz)_6_] (**1**)	88.87	95.53	94.93	94.08	82.40	84.08	89.98	7.50
[Fe_6_Dy_3_(Cl-benz)_6_] (**2**)	88.34	97.64	92.32	95.74	84.95	86.38	90.89	7.57
[Fe_6_Dy_3_(Br-benz)_6_] (**3**)	103.90	98.24	87.58	88.44	94.45	93.86	94.41	7.87
**Cluster**	**Fe1**	**Fe2**	**Fe3**	**Fe4**	**Fe5**	**Fe6**	**Θ_average_**	**av/angle**
[Fe_6_Dy_3_(benz)_6_]	285.93	242.28	312.48	285.93	242.28	312.48	280.23	11.68
[Fe_6_Dy_3_(F-benz)_6_] (**1**)	296.14	328.81	290.96	308.74	282.49	277.78	297.49	12.40
[Fe_6_Dy_3_(Cl-benz)_6_] (**2**)	288.01	327.86	284.71	313.22	287.25	280.93	296.99	12.37
[Fe_6_Dy_3_(Br-benz)_6_] (**3**)	355.97	309.49	278.26	297.38	329.02	286.95	309.51	12.90

The Σ values were found to increase stepwise from the unsubstituted Fe_6_Dy_3_ cluster to the 4-bromobenzoate cluster **3** by 8.7%. While the Σ values for the unsubstituted compound were found to be 80.3°-92.8°, these values increase and widen by substituting the benzoate, and the values are found to be 82.4°-103.9°. These values are similar to the ones reported for mononuclear octahedrally coordinated high-spin Fe(III) systems (Halcrow, 2011). This distortion from the octahedral environment leads to an average deviation of 7–8° for each of the expected 90° angles within **1**-**3**. The average Σ values within one cluster were found to be similar for the isostructural clusters **1** and **2**, where the average only changes by <1°. Cluster **3** shows that the average value is higher due to the high distortion around Fe1. Interestingly, Fe6, the Fe(III) center with the benzoate distortion, exhibits a smaller and a more close to average distortion from the octahedral environment in comparison to Fe1.

A similar trend of stepwise increase is observed for the Θ values, from an average angle of 280° for the unsubstituted Fe_6_Dy_3_ cluster, to 297° in **1** and **2** to the highest deviation of 309° within **3**, which is again an increase by 10.4% from the unsubstituted cluster to **3**. The Θ values vary between 278° and 355° (242°-312° for the unsubstituted Fe_6_Dy_3_) with similar average values for **1** and **2**, while **3** shows again a greater distortion for all Fe(III) centers. Fe1 within **3** exhibits the highest deviation from an octahedral environment. This shows that the deviation for each of the 24 torsion angles averages to 11°-13°, which highlights that the system is far from being trigonal prismatic and can be better described as a distorted octahedron. For comparison, Θ values in mononuclear and octahedrally coordinated high-spin Fe(III) coordination complexes (Halcrow, 2011) as well as Jahn-Teller active Mn(III) high-spin complexes (Gildea et al., 2014; Kühne et al., 2020) were found to be 130°-230°. The angles within **1**-**3** are slightly higher than these values but are indicative of an octahedral environment.

### SHAPE Analysis of Fe(III) and Dy(III) Ions

The unsubstituted cluster, [Fe_6_Ln_3_(μ-OMe)_9_(vanox)_6_(benz)_6_] (Kühne et al., 2016), exhibits antiferromagnetic exchange between the Fe-Fe centers. Therefore, the main SMM behavior arises from the three anisotropic Dy(III) ions. The magnetic behavior of 4f-ions is related to the interaction between the single-ion electron density and the crystal field environment (Rinehart and Long, 2011; Sorace et al., 2011; Feltham and Brooker, 2014). The coordination geometry can be analyzed using the software SHAPE (Llunell et al., 2013). For SMMs containing the oblate Dy(III), the square antiprismatic (SAP) configuration was found to be a good geometry to support SMM properties, especially for mononuclear Ln-SMMs where small changes in the coordination environment (Rinehart and Long, 2011; Sorace et al., 2011; Feltham and Brooker, 2014), ligand field strength and the complicated inter-molecular interactions could regulate the anisotropy and tune the magnetic dynamics of the mononuclear SMM systems (Neese and Pantazis, 2011). In order to describe the geometries of the lanthanide ions within the structures, they were analyzed using the software SHAPE (Llunell et al., 2013), which evaluates each geometry and assesses how far it deviates from an idealized polyhedron with zero being ideal. Table 3 summarizes the best geometry for the octacoordinated Dy(III) ions as well as the deviation from the square antiprism. For the Dy(III) centers within **1** and **2**, there are two Dy(III) centers best described as having a triangular dodecahedral environment, which is a common geometry found in multinuclear SMMs (Langley et al., 2019). The other Dy(III) center, Dy2 in both clusters, is closer to a square antiprismatic environment. This behavior is not observed in **3**, where all Dy(III) centers show small deviations from the triangular dodecahedral geometry, which is similar to that of the unsubstituted Fe_6_Dy_3_ cluster.

**Table 3:** SHAPE analysis of Fe(III) ions within 1–3, highlighting the deviation from the octahedral coordination geometry, OC-6 (top) and SHAPE analysis for all Dy(III) ions (bottom), highlighting the deviation from the square antiprismatic (SAPR) and the triangular dodecahedral (TDD) environment.

	**Fe1**	**Fe2**	**Fe3**	**Fe4**	**Fe5**	**Fe6**	**Av**
[Fe_6_Dy_3_(benz)_6_]	1.86	1.37	2.10	2.10	1.37	1.86	1.78
[Fe_6_Dy_3_(F-benz)_6_] (**1**)	2.03	2.24	2.01	2.14	1.70	1.68	1.97
[Fe_6_Dy_3_(Cl-benz)_6_] (**2**)	1.91	2.25	1.90	2.18	1.78	1.71	1.96
[Fe_6_Dy_3_(Br-benz)_6_] (**3**)	2.77	2.19	1.77	1.93	2.30	1.98	2.16
	**Dy1**		**Dy2**		**Dy3**	
	**SAPR**	**TDD**	**SAPR**	**TDD**	**SAPR**	**TDD**
[Fe_6_Dy_3_(benz)_6_]		3.77	1.60	2.52	1.75	2.52	1.74
[Fe_6_Dy_3_(F-benz)_6_] (**1**)		4.40	2.07	1.74	1.93	3.34	1.66
[Fe_6_Dy_3_(Cl-benz)_6_] (**2**)		3.73	1.87	1.79	1.98	3.27	1.72
[Fe_6_Dy_3_(Br-benz)_6_] (**3**)		4.24	1.99	2.09	1.60	3.47	1.73

The six “best” suggested geometries for the hexacoordinated Fe(III) ions are given in Table 3. This clearly shows that all Fe(III) ions exhibit a small deviation from an octahedral environment even though the angular distortion Σ and the torsional distortion Θ seem rather large. The SHAPE analysis clearly highlights that the Fe(III) centers within the unsubstituted Fe_6_Dy_3_ cluster exhibit smaller deviations from octahedral than within the clusters with the *para*-substituted benzoates. Trigonal prismatic coordination geometry is the second-best choice with an average deviation of 9.97% for **1** and 10.18% for **2** and 9.88% for **3**. As expected, within **3**, the Fe(III) center with the largest deviation from an octahedron, Fe1, shows a smaller deviation from a trigonal prism than the other Fe(III) centers.

### Packing Arrangement

In the solid state, **1** and **2** exhibit a similar packing arrangement within the crystal lattice as expected for isomorphous clusters, such that the discrete molecules are arranged in layers which are slightly tilted out of the *ab*-plane (Figure S3). It is not possible for cluster **3**, with the bigger bromo substituent, to pack in a similar way (Figure S4) as observed within **1** and **2**. The disarray of the coordinated 4-bromobenzoates does not allow the molecules to form sheets and layers like within **1** and **2**, but leads to a packing arrangement with slightly shifted molecules. The packing arrangement of all clusters shows that there are intermolecular short contacts between the neighboring molecules, formed by hydrogen bonds to the electronegative halides. These H^..^X hydrogen bonds within **1**-**3** (Figure S5) lead to the formation of a 1D chain of molecules along one preferred axis. This seems to be the dominating packing factor, which might be the reason for the change of the coordination mode of one benzoate ligand observed in **3**.

In addition to the H^..^X bonds, two substituted benzoate ligands of adjacent molecules of **1** and **2** are in close proximity to each other (Figure S6), leading to sheets of molecules within the *ab*-plane. This is not possible for **3** due to the disorder of benzoate ligands, where the steric demand of the intramolecular hydrogen bond of the unidentate bromobenzoate, Br6 (Figure 2), forces the latter out of the Fe_6_Dy_3_ plane by 39° and therefore it cannot form short contacts to neighboring molecules.

Although **3**, with one of its benzoates being monodentate, is clearly not isostructural to **1** and **2**, and its unit cell parameters are also different, there still seems to be a relationship between these crystal structures. Transformation of the unit cell of **2** (Table A1), using the matrix [1/2 1/2 0 1 −1 0 0 0 −1] results in a non-conventional cell with parameters a = 16.308 Å, b = 19.724 Å, c = 22.941 Å, α = 90.06°, β = 109.83°, and γ = 91.56°, which shows a more obvious relationship to the unit cell of **3**, and in which the rings would be nearly parallel to the new *ab* plane, as is the case in **3**. It is possible to imagine that the partial disconnection of the bromobenzoate then leads to a shearing which brings the three angles into line with those for the unit cell of **3**.

### Magnetic Properties

#### DC Magnetic Properties

**Figure 3:**
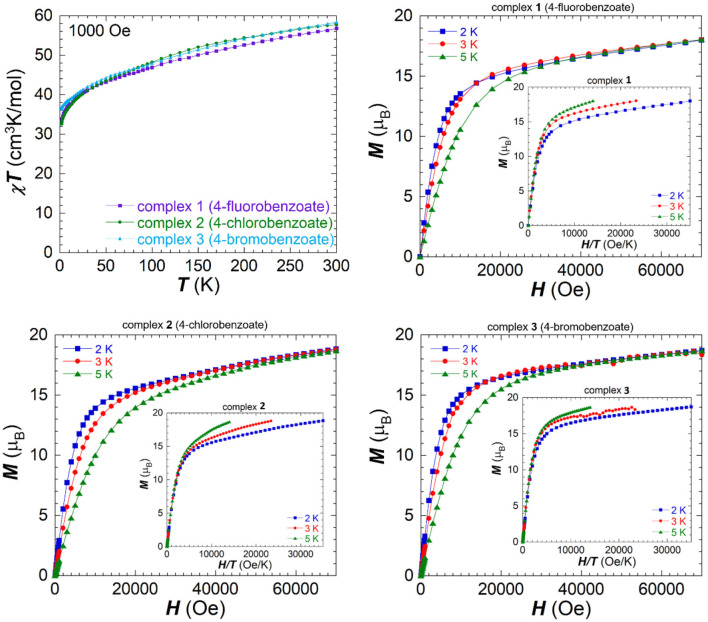
Temperature dependence of the χ_M_T product measured under an applied field of 1,000 Oe for **1**-**3** (top left), field dependence of magnetization for **1** (top right), **2** (bottom left), and **3** (bottom right) measured between 0 and 7 T at different temperatures and the reduced magnetization as inset for each.

The direct current (dc) magnetic properties were studied on freshly filtered crystalline samples (in order to avoid lattice solvent loss) in a temperature range 1.9–300 K under an applied field of 1,000 Oe and are shown as χ_*M*_*T* vs. *T* plots in Figure 3. **1**-**3** show a similar behavior to the unsubstituted cluster with a steady decrease on lowering the temperature. The χ_*M*_*T* value for each compound measured at room temperature is lower than the expected spin-only value of 68.76 cm^3^K/mol for six non-interacting Fe(III) ions and three non-interacting Dy(III) ions. The obtained values at room temperature are 56.77 cm^3^K/mol for **1** (fluorobenzoate), 57.72 cm^3^K/mol for **2** (chlorobenzoate) and 58.39 cm^3^K/mol for **3** (bromobenzoate), which is in good agreement with the unsubstituted Fe_6_Dy_3_ where a χ_*M*_*T* value of 58.8 cm^3^K/mol was observed at room temperature (Kühne et al., 2016). The data show a similar curvature for all compounds and initially decrease slowly in an almost linear fashion upon lowering the temperature to 30 K. Below this temperature the curves bend downwards and reach their minimal values at 1.9 K of 33.25 cm^3^K/mol for **1** (32.73 cm^3^K/mol for **2** and 36.89 cm^3^K/mol for **3**), indicating the presence of dominant antiferromagnetic interactions within the three Fe(III)_2_ dinuclear units. The slow and steady decrease in χ_*M*_*T* values from room temperature on, highlights the dominating antiferromagnetic coupling of the dimeric Fe_2_ units within the ring structure, which is present even at room temperature. The arrangement of these dimeric Fe(III) units in a cyclic cluster can lower the expected χ_*M*_*T* value due to the spin arrangement within the ring.

Field dependence of the magnetization was measured between 2.0 and 5.0 K and the plots are shown in Figure 3, as *M* vs. *H* curves. The lack of saturation in the magnetization values for all clusters indicates the presence of magnetic anisotropy and/or low-lying excited states. The values of the isotherms rapidly increase in all cases at low field, before following a more gradual linear increase after 1.4 T. None of the compounds show saturation of magnetization up to 7.0 T at a temperature of 2.0 K and these values of 18.0 μ_B_ for **1** (18.84 μ_B_ for **2** and 18.71 μ_B_ for **3**) are lower than the expected value for six non-interacting or ferromagnetically coupled Fe(III) centers and three Dy(III) centers. The first derivatives of the magnetization isotherms with respect to the applied field are shown in Figure S7, ESI, since such plots can be indicative of a transition between two different magnetic states (Kühne et al., 2014), or a toroidal behavior (Novitchi et al., 2012), but no maxima were observed in these plots.

The reduced magnetization curves plotted as *M* vs. *H/T* (Figure 3, inset) show at lower fields, up to 1,000 Oe (0.1 T), only small deviations from an almost perfect superposition of the three isotherms on to one master curve, which is indicative of an isotropic system within this range. On increasing the magnetic field strength, there is an increased deviation from this superposition, compatible with the presence of anisotropy and/or low-lying excited states within **1**-**3**.

#### AC Magnetic Properties

Given that all χ_*M*_*T* vs. *T* and *M* vs. *H* curves have similar shapes with similar slopes, it is likely that the coupling between the metal centers should be in a similar range. This enables us to compare **1**-**3** to see the effect of the different *para*-substituents on the dynamic magnetic behavior using ac susceptibility measurements, which previously showed slow relaxation and thus likely SMM behavior for the unsubstituted Fe_6_Dy_3_ cluster (Kühne et al., 2016). As a means to suppress any quantum tunneling of the magnetization (QTM), frequency-dependent ac susceptibility was measured at 1.8 K for all clusters with applied fields between 0 Oe and 3,000 Oe (Figure S8). The in-phase and out-of-phase ac susceptibility measurements show field-dependent signals for all compounds. Although **1** and **2** showed rather similar behavior in the dc magnetic properties, their behavior in the ac component is distinctly different. Only **2** shows weak frequency-dependent behavior in the in phase and out-of-phase ac susceptibility under zero applied field, but without visible maxima within the measured parameter set. On application of higher dc fields, it is possible to suppress the quantum tunneling component within **1**-**3** with the maxima in the out-of-phase component shifted to lower frequencies. At fields higher than 2,500 Oe, the frequency-dependent maxima in the out-of-phase susceptibility start to become very broad and/or vanish (Figure S8). The optimum dc fields where the quantum tunneling is the smallest are 1,500 Oe for **1**, 2,000 Oe for **2** and 1,000 Oe for **3**.

**Figure 4:**
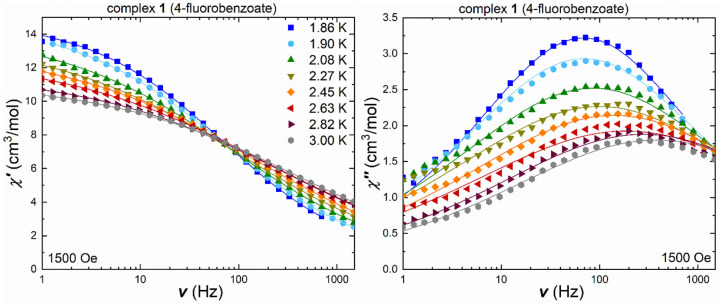
Frequency-dependent in-phase (left) and out-of-phase susceptibility (right) under an applied field of 1,500 Oe for **1** in a temperature range 1.8–3.0 K [the lines are the best fit using CCFit (Reta and Chilton, 2019)].

For the fluorobenzoate cluster **1**, the ac measurements under an applied field of 1,500 Oe, show frequency-dependent in-phase and out-of-phase signals (Figure 4), with the temperature-shifting maxima in the out-of-phase component. The Cole-Cole plots (Figure S9) were used to extract the τ and α values (Table S5) and fit the in-phase and out-of-phase susceptibility data (Figure 4) by using a generalized Debye model.

The obtained α-values are around 0.6 in the higher temperature range of 2.26–3.0 K, indicating a broad distribution of relaxation pathways most likely involving more than one relaxation process. For a single relaxation process, the Cole-Cole plot would show a perfect semi-circular profile leading to an α-value of zero. For **1**, the high deviation from this behavior is expected given the very broad maxima which spread over almost the whole frequency range. In order to check for secondary relaxation processes, especially at higher temperatures, the in-phase and out-of-phase susceptibilities were also measured in terms of their temperature-dependency under a variety of frequencies (Figure S10). The out-of-phase susceptibility component clearly highlights that the magnetic relaxation processes are limited to temperatures below 10 K, and that no secondary relaxation processes are expected beyond this temperature, since the out-of-phase susceptibility is now at zero. The temperature-dependent in-phase susceptibility (Figure S10) clearly exhibits a change in slope at low temperatures, from negative for small frequencies (1–100 Hz) to positive at frequencies beyond 200 Hz, which is indicative of multiple relaxation processes.

**Figure 5:**
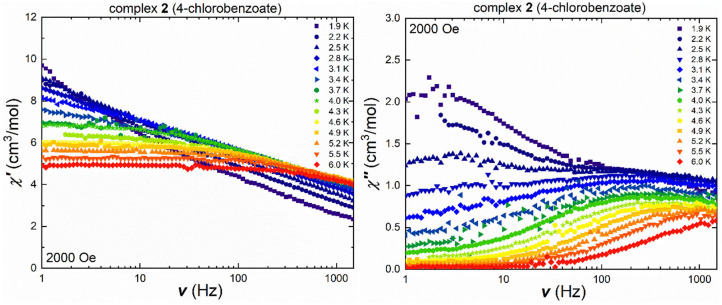
Frequency-dependent in-phase (left) and out-of-phase susceptibility (right) under an applied field of 2,000 Oe of **2** in a temperature range between 1.9 and 6.0 K.

The isostructural chlorobenzoate cluster **2** shows frequency-dependent in-phase and out-of-phase signals in the ac susceptibility measurements under an applied field of 2,000 Oe, (Figure 5), with the temperature-dependent maxima in the out-of-phase component. The maxima located at low temperatures and small frequencies appear to be frequency-independent and only start shifting at temperatures beyond 3.1 K. The Cole-Cole plot of the 2.5 K ac susceptibility data clearly shows the presence of two relaxation processes (Figure S11) and these were fitted using CCFit (Reta and Chilton, 2019) highlighting that a second process is thermally activated while the frequency-independent QTM process becomes less dominant. This is more pronounced in the temperature-dependent out-of-phase susceptibility (Figure S12), where at small frequencies and temperatures, a crossover point at 2.5 K can be observed. While the out-of-phase susceptibility curves at low frequencies display a rather sharp profile, without visible maxima, the curves become more broad at frequencies beyond 40 Hz, and no clear maxima are found due to the broadened curves, which indicates secondary magnetic relaxation processes over the measured temperature range. On the other hand, the shift of the frequency- and temperature-dependent signals at temperatures higher than 2.5 K in the out-of-phase susceptibility is easier to detect in Figure 5 due to the logarithmic x-axis. Cole-Cole plots (Figure S11) were used in the measured temperature range between 3.1 and 6.0 K, to calculate the τ and α values (Table S6). The α values decrease from 0.6 at 3.4 K to 0.2 at 5.5 K, highlighting that even at 3.4 K, the relaxation pathway is most likely a combination of several magnetic relaxation processes.

**Figure 6:**
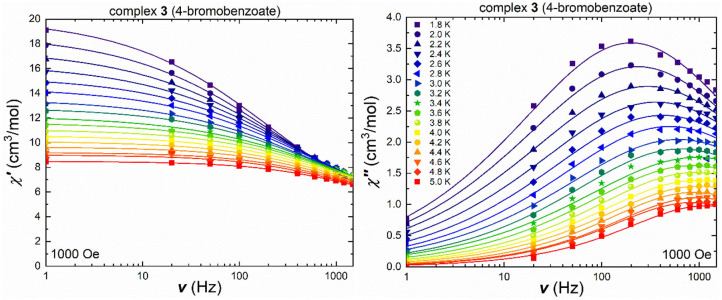
Frequency-dependent in-phase (left) and out-of-phase susceptibility (right) under an applied field of 1,000 Oe for **3** in a temperature range between 1.8 and 5.0 K [the lines show the best fit using CCFit (Reta and Chilton, 2019)].

Cluster **3**, containing the bromobenzoate ligand, which led to the structural change in coordination mode, shows frequency-dependent in-phase and out-of-phase signals in the ac measurements under an applied field of 1,000 Oe (Figure 6), as well as without an externally applied magnetic field (Figure S13) with temperature-dependent maxima in the out-of-phase component over the measured temperature range up to 5.0 K. The in-phase and out-of-phase susceptibilities were also measured in terms of their temperature-dependency under a variety of frequencies (Figures S14, S15), in order to check for secondary relaxation processes at higher temperatures and these were found to be absent. The Cole-Cole plots (Figure S1) of the ac susceptibility data at 1,000 Oe were used to extract the τ and α values (Table S7). The obtained α-values decrease on increasing the temperature from 0.5 at 1.8 K to 0.3 at 5.0 K, indicating a narrower distribution of relaxation processes and possibly corresponding to only one pathway.

**Figure 7:**
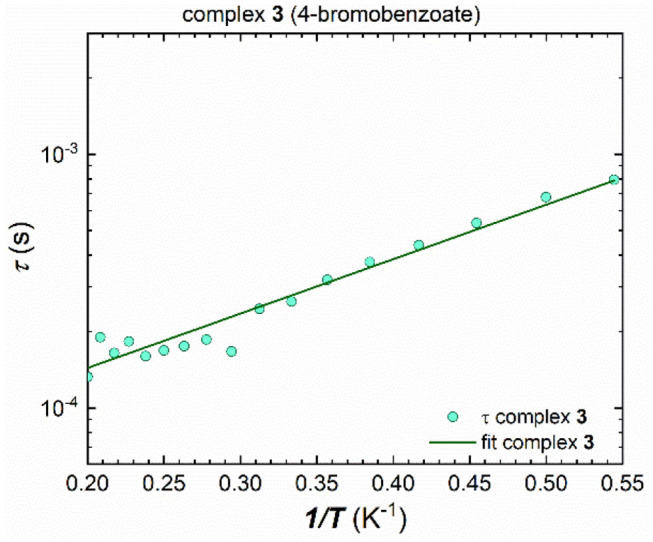
Extracted τ values plotted against 1/T for **3**, fitted to an Arrhenius law over the whole measured temperature range, leading to U_eff_ = 4.9 K and τ_0_ = 5.2^.^10^−5^ s.

The energy barrier for spin reversal for **1**-**3** was fitted using the extracted τ values from the Cole-Cole plots which are then plotted against the reciprocal temperature, 1 /T. In contrast to **1** and **2**, cluster **3** could be fitted using the Arrhenius law for all τ values (Figure 7), since a distinct linear trend can be observed by plotting τ against 1/T, which leads to U_eff_ = 4.9 K and τ_0_ = 5.2^.^10^−5^ s. Considering not just thermally induced relaxation processes, but also other relaxation mechanisms such as Raman and QTM (which seems to be fully suppressed) processes, and fitting combinations or all of them simultaneously, using Equation (1), does not lead to any improvement (Figure S17).

$$\begin{array}{l} \tau^{-1}=\tau_{0}^{-1}exp\left(-\frac{U_{eff}}{T}\right)+CT^{n}+\tau_{QTM}^{-1} \end{array}$$

**Figure 8:**
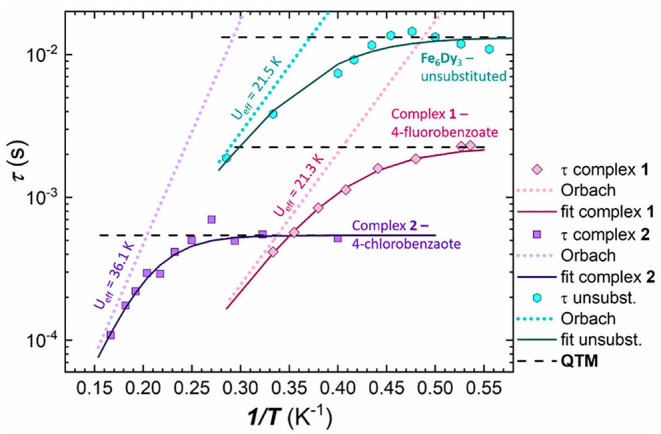
Extracted τ values plotted against 1/T for 1 (4-fluorobenzoate; pink) 2 (4-chlorobenzoate; purple) and the unsubstituted Fe_6_Dy_3_ cluster (turquoise), with the respective simultaneous Orbach-QTM fit (solid line) (Orbach process—dotted lines; QTM factor—dashed black lines).

**1** and **2** show a noticeably different behavior in their magnetic relaxation mechanism compared to **3** (Figure 8). It is not possible to fit all data points of the τ values with one single slope over the measured temperature range. It has been shown in the literature, that by ignoring the overall slope of the extracted τ values and constraining a linear regime at higher temperatures, in which the data could be fitted to the Arrhenius law, can lead to an erroneously large *U*_eff_ value (Lampropoulos et al., 2009). Therefore, the spin relaxation was simultaneously fitted using Equation (1) (Reta and Chilton, 2019), considering all three possible relaxation processes, including a tunneling process, QTM, at low temperatures, followed by a Raman and an Orbach processes. This approach was only applied to **2** (Figure S18), since more data in form of τ values are available for **2** than for **1** and it is thus not possible to get a reasonable fit for the τ values of **1** as a result of the poor data:parameter ratio.

The best simultaneous fit of Equation (1), for **2**, shows that the magnetic relaxation is dominated by quantum tunneling at low temperatures, followed by an Orbach process at higher temperatures as indicated by the almost linear dependence. Thus the relaxation process of **1** was fitted according to this outcome from the isostructural cluster **2** (i.e. without a Raman process), in order to avoid overparameterization. In case of **1**, the quantum tunneling parameter was determined as τ_QTM_ = 2.3^.^10^−3^ s, which includes only the points at very low temperatures up to 2.0 K, whilst for **2** the QTM process is the dominant process up to 3.4 K with a faster τ_QTM_ factor of 5.4^.^10^−3^ s. The exponential fit of the Orbach processes leads to energy barriers of U_eff_ = 21.3 K for **1** and U_eff_ = 36.1 K for **2**, with τ_0_ = 4.1^.^10^−7^ s for **1** and τ_0_ = 3.4^.^10^−7^ s for **2**, respectively, indicating similar relaxation processes and energy barriers for both.

In order to compare this result with the unsubstituted cluster, we used the published ac data (Kühne et al., 2016) and ran new simulations to fit the in-phase and out-of-phase susceptibilities of the Cole-Cole plot (Figure S19 and Table S8) using CCFit (Reta and Chilton, 2019). The energy barrier was then fitted using the extracted τ values and Equation (1) (Figure S20) in a similar way as for **1** and **2**, leading to an Orbach process with an energy barrier of U_eff_ = 21.5 K and τ_0_ = 4.5^.^10^−6^ s and a QTM process below 2.2 K. This illustrates that the fluorosubstituted cluster **1** shows a similar behavior to that of the unsubstituted Fe_6_Dy_3_ cluster (Figure 8), where the complete fit of both clusters exhibits a nearly parallel performance, leading to almost identical energy barriers in the Orbach component of U_eff_ = 21.5 K for the unsubstituted cluster and U_eff_ = 21.3 K for **1**. This is reasonable due to the similarity of the 4-fluorobenzoic acid and the benzoic acid in terms of their acidity.

In 2011, Mereacre et al. established a correlation between the energy barrier U_eff_ and the Hammett parameters of the coordinated substituted benzoic acids in a butterfly Fe_2_Dy_2_ compound (Mereacre et al., 2011). In these compounds, each Dy(III) is coordinated by four carboxylate oxygens that are all well-aligned around the Dy(III) centers on the direction of the local Dy anisotropy axis. In the system shown here, each Dy(III) ion is only ligated by two carboxylate oxygens that bridge to an Fe(III) center on either side, which therefore can be seen as *cis* to each other. The pk_a_ value of benzoic acid (4.19) is similar to that of 4-fluorobenzoic acid (4.15) and therefore the Hammett parameters for both are similar too, with 4-fluorobenzoate = −0.03 (Hansch et al., 1991). By increasing the strength of the acid toward 4-chlorobenzoate (pk_a_ = 3.98; = +0.19) (Hansch et al., 1991) an increase in the energy barrier, U_eff_ = 36.1 K for **2** was observed. Since the Hammett parameter of 4-bromobenzoate (= +0.25) (Hansch et al., 1991) is bigger than that of 4-chlorobenzoate, while the pk_a_ of both acids are in a similar range (3.98 for Cl vs. 3.97 for Br), a comparison between **3** and the other compounds should show whether the trend of the increase of U_eff_ tracks the Hammett parameters or the pk_a_ values of the ligand. Unfortunately, as cluster **3** features a different structure, a direct comparison cannot be made.

## Conclusions

We report the structural and magnetic properties of three nonanuclear Fe_6_Dy_3_ cyclic coordination clusters with para-halosubstituted benzoate ligands. **1** and **2**, the fluoro- and chlorobenzoate clusters, are isostructural and can be regarded as the cyclisation of three {Fe_2_Ln} units. In the case of the bromo substituent within **3**, we can no longer use this model, since one of its benzoates is monodentate: it does not bridge between one Fe(III) and one Dy(III) ion anymore, but coordinates only to a single Dy(III) ion. The change in pK_a_/Hammett constants for these benzoic acids on moving from *para*-Cl to *para*-Br results in this “carboxylate shift” from the bridging to monodentate coordination. A second influence is the size of the halo-substituent forcing the benzoate ligands of the cyclic coordination clusters out of the metal plane in order to keep the packing arrangement similar. This results in one oxygen of a 4-bromobenzoate carboxylate group maintaining its coordination to the Dy(III) ion, but with the other oxygen now losing its grip on the neighboring Fe(III) ion and becoming just a hydrogen-bond acceptor; this switch to monodentate coordination by the carboxylate requires the ligation of a methanol molecule to the Fe(III) to maintain its 6 coordination. This coordinative flexibility opens possibilities for future applications such as MRI contrast agents or catalytic activities.

These considerations are also relevant to the dynamic magnetic properties within these antiferromagnetically coupled coordination clusters. Whereas, **1** and **2** show similar behavior in their magnetic relaxation processes, with a combination of QTM and Orbach mechanisms, the QTM part can be completely suppressed within **3** by application of a small dc field. Furthermore, **1**, which has the *para*-fluorobenzoate as a ligand, shows behavior similar to that of the unsubstituted Fe_6_Dy_3_ cluster, as shown by the complete fit of the data for both clusters being almost parallel. The energy barrier, U_eff_, increases significantly on going to the *para*-chlorobenzoate within **2** from U_eff_ = 21.3 K for **1** to U_eff_ = 36.1 K for **2**. This reflects the changes in pK_a_ and Hammett constants, but a clear trend cannot be identified due to the structural break observed on going to the 4-bromobenzoate within **3**.

## Materials and Methods

### Materials and Instrumentation

All chemicals and solvents unless otherwise stated were of reagent grade and used as received from commercial suppliers. They were used without further purification or drying. Dy(NO_3_)${\;}_{3}^{.}$6H_2_O was synthesized from Dy_2_O_3_ by treatment with nitric acid, HNO_3_. *o*-Vanillinoxime (H_2_vanox) was prepared according to the literature (Hewitt et al., 2009). Elemental analysis (C, H, N) was recorded on dried polycrystalline samples using an Exeter Analytical CE-440 CHN analyzer. Infrared spectra were recorded using a Bruker Alpha Platinum ATR (attenuated total reflection) spectrometer. PXRD pattern were recorded on dried samples using a Stoe STADI-4 XRD diffractometer equipped with a Cu-Kα source.

### Synthesis of Compounds 1-3

The synthesis of **1**-**3** is straightforward, and the compounds were recovered in varying yields. All reactions are modified versions of the published work on the unsubstituted benzoate Fe_6_Ln_3_ compounds (Kühne et al., 2016). The elemental analysis was performed on dried polycrystalline samples. Due to the volatility of the methanol molecules within the crystal lattice, the elemental analysis was fitted using water molecules in the crystal lattice replacing the methanol molecules. The IR spectra of **2** and **3** are shown in Figure S21, and highlight the structural similarities of the clusters, including the carboxylate C-O stretching bands [~1,540 cm^−1^ (antisymmetric) and ~1,410 cm^−1^ (symmetric)] and the oxime bands (C=N ~1,590 cm^−1^). No differences resulting from a monodentate 4-bromobenzoate in **3** are found in the IR spectra. PXRD patterns of **2** and **3** were recorded on dried samples (Figure S23), highlighting the presence of the main peaks in the correct positions. The relative intensities of the peaks show some differences with the predicted pattern likely caused by the loss of solvent. However, despite this the extracted unit cell parameters are in good agreement with the single crystal data.

Compound **1**: [Fe_6_Dy_3_(μ-OMe)_9_(vanox)_6_(F-benz)_6_]^.^12MeOH^.^H_2_O: H_2_vanox (0.4 mmol; 0.066 g) and NaOMe (1.0 mmol; 0.054 g) were dissolved in 10 ml MeOH and were added to a solution of ${\text{FeCl}}_{2}^{.}$4H_2_O (0.2 mmol; 0.040 g), Dy(NO_3_)${\;}_{3}^{.}$6H_2_O (0.2 mmol; 0.092 g) and 4-fluorobenzoic acid (0.4 mmol; 0.056 g) in 10 ml MeOH. The dark red, almost black, solution was stirred for 10 min at room temperature, and left to stand for crystallization without filtration. After 1 week, **1** crystallized as dark red-black blocks suitable for single crystal X-ray analysis.

Yield of [Fe_6_Dy_3_(μ-OMe)_9_(vanox)_6_(F-benz)_6_]^.^12MeOH^.^ H_2_O: 55 mg (50% related to Fe)

Elemental analysis for [C_99_H_93_Fe_6_Dy_3_N_6_O_39_F_6_]^.^10.6H_2_O (%): calculated: C: 38.13, H: 3.69; N: 2.69; found: C: 38.11; H: 3.67; N: 2.71.

IR (KBr):/cm^−1^ = 3,441 (m), 2,822 (w), 1,606 (s), 1,591 (s), 1,543 (s), 1,508 (w), 1,459 (s), 1,439 (m), 1,410 (s), 1,354 (w), 1,269 (m), 1,239 (w), 1,221 (m), 1,153 (m), 1,094 (w), 1,056 (s), 965 (m), 856 (m), 783 (m), 764 (m), 737 (w), 691 (w), 657 (m), 618 (m), 555 (w), 500 (w), 461 (m).

Compound **2**: [Fe_6_Dy_3_(μ-OMe)_9_(vanox)_6_(Cl-benz)_6_]^.^ 13MeOH: H_2_vanox (0.4 mmol; 0.066 g) and NaOMe (1.5 mmol; 0.081 g) were dissolved in 10 ml MeOH and were added to a solution of ${\text{FeCl}}_{2}^{.}$4H_2_O (0.2 mmol; 0.040 g), Dy(NO_3_)${\;}_{3}^{.}$6H_2_O (0.2 mmol; 0.092 g) and 4-chlorobenzoic acid (0.4 mmol; 0.063 g) in 10 ml MeOH. The dark red, almost black, solution was stirred for 10 min at room temperature, and left to stand for crystallization without filtration. After 1 week, **2** crystallized as dark red-black blocks suitable for single crystal X-ray analysis.

Yield of [Fe_6_Dy_3_(μ-OMe)_9_(vanox)_6_(Cl-benz)_6_]^.^13MeOH: 51 mg (44% related to Fe)

Elemental analysis for [C_99_H_93_Fe_6_Dy_3_N_6_O_39_Cl_6_]^.^4.6MeOH^.^ 5.9H_2_O (%): calculated: C: 37.94, H: 3.78; N: 2.56; found: C: 37.73; H: 3.57; N: 2.35.

IR (KBr):/cm^−1^ = 3,439 (m), 2,991 (w), 2,927 (m), 2,821 (m), 1,592 (s), 1,537 (s), 1,459 (s), 1,407 (s), 1,268 (s), 1,221 (s), 1,171 (m), 1,093 (s), 1,056 (s), 964 (s), 853 (m), 776 (m), 737 (m), 656 (s), 584 (w), 540 (m), 458 (m).

Compound **3**: [Fe_6_Dy_3_(μ-OMe)_9_(vanox)_6_(Br-benz)_6_ (MeOH)]^.^9MeOH: H_2_vanox (0.4 mmol; 0.066 g) and NaOMe (1.5 mmol; 0.081 g) were dissolved in 10 ml MeOH and were added to a solution of ${\text{FeCl}}_{2}^{.}$4H_2_O (0.2 mmol; 0.040 g), Dy(NO_3_)${\;}_{3}^{.}$6H_2_O (0.2 mmol; 0.092 g), and 4-bromobenzoic acid (0.4 mmol; 0.080 g) in 10 ml MeOH and 5 ml CH_2_Cl_2_. The dark red, almost black, solution was stirred for 10 min at room temperature, then filtered and left to stand for crystallization. After 2 weeks, **3** crystallized as dark red-black blocks suitable for single crystal X-ray analysis.

Yield of [Fe_6_Dy_3_(μ-OMe)_9_(vanox)_6_(Br-benz)_6_(MeOH)]^.^ 9MeOH: 43 mg (36% related to Fe)

Elemental analysis for [C_100_H_97_Fe_6_Dy_3_N_6_O_40_Br_6_]^.^0.9 MeOH^.^13.2H_2_O (%): calculated: C: 33.74, H: 3.56; N: 2.34; found: C: 33.52; H: 3.79; N: 2.57.

IR (KBr):/cm^−1^ = 3,437 (m), 2,943 (w), 2,822 (w), 1,588 (s), 1,537 (s), 1,459 (s), 1,438 (w), 1,406 (s), 1,356 (w), 1,270 (m), 1,222 (m), 1,171 (m), 1,057 (s), 1,013 (m), 966 (m), 854 (m), 771 (s), 736 (m), 657 (m), 572 (w), 504 (m), 460 (m).

### Single Crystal X-Ray Structure Determination

X-ray crystallography was carried out on suitable single crystals using an Oxford Supernova diffractometer (Oxford Instruments, Oxford, United Kingdom). Datasets were measured using monochromatic Cu-Kα (**2**) or Mo-Kα (**1** and **3**) radiation and corrected for absorption. The temperature was controlled with an Oxford Cryosystem instrument. All structures were solved by dual-space direct methods (SHELXT) and refined by full matrix least-squares on F2 for all data using SHELXL-2016 (Sheldrick, 2015). All hydrogen atoms were added at calculated positions and refined using a riding model. Their isotropic displacement parameters were fixed to 1.2 times the equivalent one of the parent atom. Anisotropic displacement parameters were used for all ordered non-hydrogen atoms. Crystallographic details for **1**-**3** are summarized in Table A1 and crystallographic data for the structures reported in this paper have been deposited with the Cambridge Crystallographic Data Center as supplementary publication numbers CCDC2001519-2001521.

### Magnetic Measurements

The magnetic susceptibility measurements were obtained using a Quantum Design SQUID magnetometer MPMS-XL (Quantum Design, San Diego, USA) operating between 1.8 and 300 K. DC (direct current) measurements were performed on freshly filtered polycrystalline samples to avoid loss of lattice solvent. Each sample was wrapped in a polyethylene membrane, and susceptibility data were collected at 0.1 T between 1.8 and 300 K in cooling mode. The magnetization data was collected at 2, 3, and 5 K. Diamagnetic corrections were applied to correct for contribution from the sample holder, and the inherent diamagnetism of the sample was estimated with the use of Pascal's constants. AC (alternating current) measurements were carried out in with frequencies between 1 and 1,500 Hz.

## Data Availability Statement

The datasets presented in this study can be found in online repositories. The names of the repository/repositories and accession number(s) can be found in the article/Supplementary Material. Crystallographic data have been deposited with the CCDC (Cambridge Crystallographic Data Centre) Numbers: 2001519–2001521. Copies of the data can be obtained free of charge from https://www.ccdc.cam.ac.uk/structures/.

## Author Contributions

IK and AP were responsible for the conceptualization of the project. IK was responsible for the synthesis and standard characterizations, performed the analysis of the magnetic data, and drafted the initial manuscript. CA collected and refined the single crystal data whilst IK and CA performed the structural analysis. The manuscript was written through contributions of all authors. All authors have given approval to the final version of the manuscript.

## Conflict of Interest

The authors declare that the research was conducted in the absence of any commercial or financial relationships that could be construed as a potential conflict of interest.

## Appendix

**Table A1:** Crystallographic Data for the cyclic coordination clusters **1**-**3**.

**Compound**	**Cluster 1**	**Cluster 2**	**Cluster 3**
Identification code	Fe6Dy3-F	Fe6Dy3-Cl	Fe6Dy3-Br
Empirical formula	C_111_H_143_Dy_3_F_6_Fe_6_N_6_O_52_	C_112_H_145_Cl_6_Dy_3_Fe_6_N_6_O_52_	C_109_H_133_Br_6_Dy_3_Fe_6_N_6_O_49_
Formula weight	3329.91	3442.63	3613.27
Temperature (K)	150(2)	180(2)	150(2)
Crystal system	triclinic	Triclinic	Triclinic
Space group	*P-1*	*P-1*	*P-1*
Crystal size (mm)^3^	0.36 × 0.28 × 0.11	0.17 × 0.12 × 0.07	0.37 × 0.23 × 0.19
*a* (Å)	18.4290(14)	18.8093(7)	16.9250(8)
*b* (Å)	18.9102(15)	19.2724(6)	19.7948(10)
*c* (Å)	22.7645(17)	22.9728(6)	22.4771(12)
α (°)	74.458(6)	73.406(2)	107.361(4)
β (°)	74.312(6)	72.925(3)	94.136(4)
γ (°)	61.099(5)	62.357(3)	107.204(4)
V (Å^3^)	6597.3(10)	6941.4(4)	6758.7(6)
Z	2	2	2
d_calc_ (Mg cm^–3^)	1.676	1.647	1.775
μ (mm^–1^)	2.414	15.143	4.119
Radiation	Mo-Kα	Cu-Kα	Mo-Kα
F(000)	3,350	3,462	3,570
Limiting indices	h = ±22, k = ±23, l = ±28	h = ±23, k = ±23, l = ±28	h = ±21, k = ±25, l = ±28
Reflections collected/unique	68115/17547	118864/19723	86682/19676
R(int)	0.0514	0.0677	0.0563
Data/restraints/parameters	26614/12/1584	27024/0/1487	28714/102/ 1547
GooF on F^2^	0.897	0.990	0.917
Final R indices [I > 2(I)]^a^	R_1_ = 0.0555; wR_2_ = 0.1314	R_1_ = 0.0511; wR_2_ = 0.1266	R_1_ = 0.0498; wR_2_ = 0.1182
R indices (all data)	R_1_ = 0.0838; wR_2_ = 0.1404	R_1_ = 0.0731; wR_2_ = 0.1377	R_1_ = 0.0770; wR_2_ = 0.1282
Largest diff. peak/hole (e^.^Å^–3^)	1.289/–2.625	1.277/–1.754	1.894/–1.479
CCDC number	2001519	2001520	2001521
