# Supplementary material for: The Influence of Halide Substituents on the Structural and Magnetic Properties of Fe6Dy3 Rings
Source: Front Chem. 2020 Aug 14;8:701. doi: 10.3389/fchem.2020.00701 (PMC7456939; doi:10.3389/fchem.2020.00701)
Supplement: Supplementary file 1 [file Data_Sheet_1.pdf]

Supplementary Material

Index

Supplementary **Figure 1**. Structural overlap of complex **1** containing the 4-fluorobenzoate (yellow), **2** containing the 4-chlorobenzoate (green) and complex 3 containing the 4-bromobenzoate (purple), .3

Supplementary **Figure 2**. Plane through all nine metal centers of the 4-fluorobenzoate containing complex **1**, highlighting the deviation of the benzoate rings tilted out of the plane 3

Supplementary **Table S1**. M…M distances found within complexes **1 - 3** (in Å). 4

Supplementary **Table S2**. Angles (in °) between the metal plane (derived from all six Fe(III) centers) and the benzene ring (all six carbon atoms of the ring) of the 4-X-benzoate for complexes **1 - 3** 4

Supplementary **Table S3**. Bond lengths around the Fe(III) centers within complexes **1 - 3** (in °) in comparison the unsubstituted Fe_6Dy_3 complex 5

Supplementary **Table S4**. Bond lengths around the Dy(III) centers within complexes **1 - 3** (in °) in comparison the unsubstituted Fe_6Dy_3 complex 6

Supplementary **Figure 3**. Packing arrangement of **2** along *c*-axis (left) and *a*-axis (right). 7

Supplementary **Figure 4**. Packing arrangement of **3** along *c*-axis (left) and *a*-axis (right). 7

Supplementary **Figure 5a**. Hydrogen bonds, H-F, between neighbouring molecules within compound **1** shown as turcoise dashed lines, forming chains along a within the ab-plane. 8

Supplementary **Figure 5b**. Hydrogen bonds, H-Cl, between two neighbouring molecules within compound **2** shown as turcoise dashed lines. 8

Supplementary **Figure 5c**. Hydrogen bonds, H-Br, between two neighbouring molecules within compound **3** shown as turcoise dashed lines, forming chains along the *b*-axis within the *ab*-plane. 8

Supplementary **Figure 6**. Close contacts between neighbouring 4-fluorobenzoate ligands withing complex **1** (top) and neighbouring 4-chlorobenzoate ligands withing complex 2 (bottom) 9

Supplementary **Figure 7**. The first derivatives of the magnetization with respect to the applied field dM/dH (lines are guides to the eyes) for complexes **1 - 3** at 2 K, 3 K and 5 K.....................................9

Supplementary **Figure 8**. In-phase (left) and out-of-phase ac susceptibility (right) at 1.8 K and varying field between 0 Oe and 3000 Oe for complexes **1 - 3**. 10

Supplementary **Figure 9**. Cole-Cole plot between 1.86 and 3.0 K for complex **1** measured at 1500 Oe. 11

Supplementary **Table S5**. Resulting parameters of τ and α obtained with CC-Fit for complex **1** at 1500 Oe. 11

Supplementary **Figure 10**. Temperature dependent ac susceptibility using different frequencies between 1.0 - 1488.0 Hz, for complex **1** measured under a dc field of 1500 Oe with the in-phase (left) and out-of-phase ac susceptibility (right) in a temperature range between 1.9 - 6.0 K.......................11

Supplementary **Figure 11**. Cole-Cole plot for 2.5 K (left) and in the temperature range between 3.1 - 6.0 K (right) for complex **2** measured at 2000 Oe and in-phase and out-of-phase ac susceptibility fit using CCFit^2 in a temperature range between 3.1 - 6.0 K. 12

Supplementary **Figure 12**. Temperature dependent ac susceptibility using different frequencies between 1.0 - 1488.0 Hz, for complex **2** measured under a dc field of 2000 Oe with the in-phase (left) and out-of-phase ac susceptibility (right) in a temperature range between 1.9 - 6.0 K....12

Supplementary **Table S6**. Resulting parameters of τ and α obtained with CC-Fit^2 for complex **2** at 2000 Oe. 13

Supplementary **Figure 13**. Frequency-dependent in-phase (left) and out-of-phase susceptibility (right) without applied field of complex **3** in a temperature range between 1.8 - 4.8 K 13

Supplementary **Figure 14**. Temperature dependent ac susceptibility using different frequencies between 1.0 - 1488.0 Hz, for complex **3** measured under a dc field of 1000 Oe with the in-phase (left) and out-of-phase ac susceptibility (right) in a temperature range between 1.9 - 5.0 K....14

Supplementary **Figure 15**. Temperature dependent ac susceptibility using different frequencies between 1.0 - 1488.0 Hz, for complex **3** measured without applied dc field with the in-phase (left) and out-of-phase ac susceptibility (right) in a temperature range between 1.9 - 4.8 K....14

Supplementary **Figure 16**. Cole-Cole plot between 1.8 and 5.0 K for complex **3** measured at 1000 Oe using CCFit^2 over the whole measured temperature range 15

Supplementary **Table S7**. Resulting parameters of τ and α obtained with CC-Fit^2 for complex **3** at 1000 Oe. 15

Supplementary **Figure 17**. Extracted τ values plotted against 1/T (black squares) and simultaneous Orbach-QTM fit (red line) and for Raman-QTM fit (green dashed line) for complexes **3**,....16

Supplementary **Figure 18**. Extracted τ values plotted against 1/T (black squares) for complex **1**; simultaneous Orbach-Raman-QTM fit (top left), simultaneous Orbach-Raman fit (top right), simultaneous Raman-QTM fit (bottom left) and simultaneous Orbach-QTM fit (bottom right)....17

Supplementary **Figure 19**. Frequency-dependent in-phase and out-of phase susceptibility under an external dc field of 2000 Oe (left) for the unsubstituted Fe_6Dy_3 complex in a temperature range between 1.8 - 3.5 K; Cole-Cole plots (right) between 1.8 and 3.5 K using CCFit^2 over the whole measured temperature range (right)....18

Supplementary **Table S8**. Resulting parameters of τ and α obtained with CC-Fit for the unsubstituted Fe_6Dy_3 compound. 18

Supplementary **Figure 20**. Extracted τ values plotted against 1/T (black squares) for the unsubstituted Fe_6Dy_3 complex and the simultaneous Orbach-QTM fit 19

Supplementary **Figure S21**. Comparison of Infrared (IR) spectra of compounds **2** (4-chloro, blue) and **3** (4-bromo, purple). 19

Supplementary **Figure S22**. PXRD pattern (black), fit (red), predicted peak position (blue) and difference between predicted and calculated pattern (green) for dried polycrystalline complex **2** (left) and complex **3** (right), leading to Rp = 8.93 (complex 2) and Rp = 8.14 (complex 3). 20

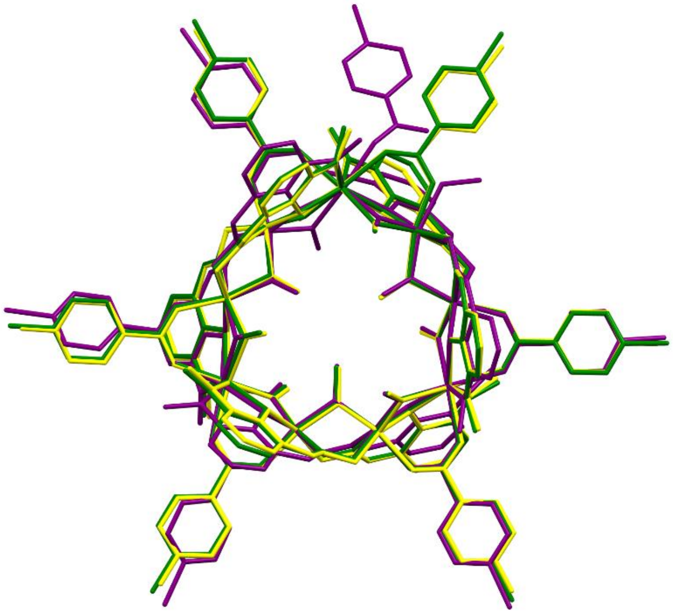

Supplementary Figure 1. Structural overlap of complex **1** containing the 4-fluorobenzoate (yellow), **2** containing the 4-chlorobenzoate (green) and complex **3** containing the 4-bromobenzoate (purple), by superposition of all nine metal centers, highlightling the discrepancy in overlap of the substituted benzoate ligands.

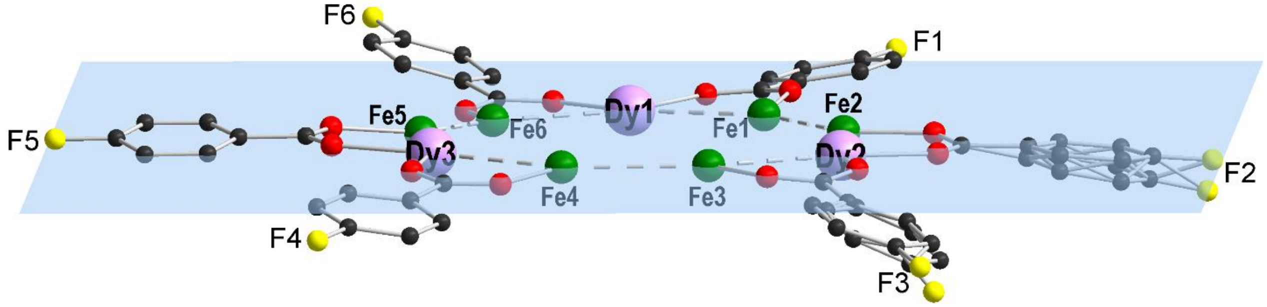

Supplementary Figure 2. Plane through all nine metal centers of the 4-fluorobenzoate containing complex **1**, highlighting the deviation of the benzoate rings tilted out of the plane (all other ligands, except the 4-halobenzoates, emitted for clarity).

Supplementary Table S1. M…M distances found within complexes **1 - 3** (in Å).

	complex **1**	complex **2**	complex **3**
Fe1-Fe2	3.271	3.288	3.282
Fe3-Fe4	3.284	3.268	3.274
Fe5-Fe6	2.273	3.276	3.247
Fe1-Dy1	3.4	3.407	3.404
Fe2-Dy2	3.411	3.403	3.412
Fe3-Dy2	3.412	3.376	3.393
Fe4-Dy3	3.407	3.414	3.391
Fe5-Dy3	3.375	3.399	3.395
Fe6-Dy1	3.398	3.423	3.487

Supplementary Table S2. Angles (in °) between the metal plane (derived from all six Fe(III) centers) and the benzene ring (all six carbon atoms of the ring) of the 4-X-benzoate for complexes **1 - 3**

formula	X1	X2	X3	X4	X5	X6
$[Fe_6Dy_3(F\text{-}benz)_6]$ (**1**)	8.98	5.25/10.67	18.82/26.30	13.56	9.21	17.78
$[Fe_6Dy_3(Cl\text{-}benz)_6]$ (**2**)	11.53	8.75	19.47	11.72	2.53	14.48
$[Fe_6Dy_3(Br\text{-}benz)_6]$ (**3**)	9.40	34.18	30.28	26.20/25.08/24.43	22.35	38.95

Supplementary Table S3. Bond lengths around the Fe(III) centers within complexes **1 - 3** (in °) in comparison the unsubstituted Fe_6Dy_3 complex
(which crystallized in the monoclinic space group *C2/c*, therefore half the molecule is generated by symmetry operations).

Fe-O/N	Fe_6Dy_3 (original)	complex **1**	complex **2**	complex **3**
	Fe1-O1 1.953(4)	Fe1-O15 1.943(5)	Fe1-O1 1.950(4)	Fe1-O1 1.934(5)
	Fe1-O10 1.965(4)	Fe1-O1 1.947(4)	Fe1-O2 2.009(4)	Fe1-O15 1.952(4)
	Fe1-O2 2.009(4)	Fe1-O2 2.008(5)	Fe1-O10 2.037(3)	Fe1-O2 2.015(4)
	Fe1-O6 2.038(4)	Fe1-O10 2.043(4)	Fe1-O15 1.954(4)	Fe1-O10 2.058(4)
	Fe1-O16 2.064(4)	Fe1-O29 2.072(5)	Fe1-O29 2.064(4)	Fe1-O29 2.070(4)
	Fe1-N1 2.110(6)	Fe1-N1 2.090(5)	Fe1-N1 2.099(4)	Fe1-N1 2.104(6)
	Fe2-O7 1.973(4)	Fe2-O3 1.947(5)	Fe2-O2 1.983(3)	Fe2-O3 1.941(4)
	Fe2-O3 1.974(4)	Fe2-O2 2.001(4)	Fe2-O3 1.960(4)	Fe2-O12 1.982(5)
	Fe2-O2 1.990(4)	Fe2-O12 2.003(4)	Fe2-O12 1.998(4)	Fe2-O2 1.990(4)
	Fe2-O17 2.059(4)	Fe2-O13 2.054(4)	Fe2-O14 2.059(3)	Fe2-O30 2.052(4)
	Fe2-O9 2.061(4)	Fe2-O30 2.054(5)	Fe2-O30 2.052(4)	Fe2-O13 2.066(4)
	Fe2-N2 2.117(5)	Fe2-N2 2.124(6)	Fe2-N2 2.117(5)	Fe2-N2 2.112(5)
	Fe3-O4 1.958(4)	Fe3-O4 1.941(5)	Fe3-O4 1.952(4)	Fe3-O4 1.941(4)
	Fe3-O13 1.983(4)	Fe3-O21 1.964(4)	Fe3-O5 1.997(4)	Fe3-O21 1.985(5)
	Fe3-O5 2.008(3)	Fe3-O5 2.007(5)	Fe3-O16 2.079(4)	Fe3-O5 1.994(4)
	Fe3-O12 2.061(4)	Fe3-O33 2.053(5)	Fe3-O21 1.962(4)	Fe3-O16 2.048(4)
	Fe3-O20 2.069(4)	Fe3-O16 2.071(4)	Fe3-O33 2.048(4)	Fe3-O33 2.059(4)
	Fe3-N3 2.109(5)	Fe3-N3 2.103(6)	Fe3-N3 2.110(5)	Fe3-N3 2.115(5)
	Fe3’-O4’ 1.958(4)	Fe4-O6 1.954(5)	Fe4-O5 2.020(4)	Fe4-O6 1.953(4)
	Fe3’-O13’ 1.983(4)	Fe4-O17 1.959(5)	Fe4-O6 1.947(4)	Fe4-O18 1.955(5)
	Fe3’-O5 2.008(3)	Fe4-O5 2.025(4)	Fe4-O18 1.966(4)	Fe4-O5 1.998(4)
	Fe3’-O12’ 2.061(4)	Fe4-O19 2.049(5)	Fe4-O19 2.048(4)	Fe4-O19 2.029(5)
	Fe3’-O20’ 2.069(4)	Fe4-O34 2.074(5)	Fe4-O34 2.070(4)	Fe4-O34 2.068(5)
	Fe3’-N3’ 2.109(5)	Fe4-N4 2.119(6)	Fe4-N4 2.113(5)	Fe4-N4 2.120(6)
	Fe2’-O7’ 1.973(4)	Fe5-O8 1.960(5)	Fe5-O8 1.993(3)	Fe5-O7 1.953(4)
	Fe2’-O3’ 1.974(4)	Fe5-O27 1.963(5)	Fe5-O7 1.952(4)	Fe5-O27 1.962(5)
	Fe2’-O2’ 1.990(4)	Fe5-O7 1.989(4)	Fe5-O22 2.024(4)	Fe5-O8 2.008(4)
	Fe2’-O17’ 2.059(4)	Fe5-O22 2.015(5)	Fe5-O27 1.955(4)	Fe5-O22 2.044(4)
	Fe2’-O9’ 2.061(4)	Fe5-O37 2.060(5)	Fe5-O37 2.063(4)	Fe5-O37 2.078(4)
	Fe2’-N2’ 2.117(5)	Fe5-N5 2.113(6)	Fe5-N5 2.099(5)	Fe5-N5 2.094(5)
	Fe1’-O1’ 1.953(4)	Fe6-O9 1.956(4)	Fe6-O8 1.985(4)	Fe6-O9 1.947(5)
	Fe1’-O10’ 1.965(4)	Fe6-O7 1.978(5)	Fe6-O9 1.961(4)	Fe6-O24 1.970(5)
	Fe1’-O2’ 2.009(4)	Fe6-O24 1.988(4)	Fe6-O24 1.982(4)	Fe6-O8 1.985(4)
	Fe1’-O6’ 2.038(4)	Fe6-O25 2.028(4)	Fe6-O25 2.041(3)	Fe6-O40 2.057(5)
	Fe1’-O16’ 2.064(4)	Fe6-O38 2.050(5)	Fe6-O38 2.045(4)	Fe6-O25 2.087(4)
	Fe1’-N1’ 2.110(6)	Fe6-N6 2.128(5)	Fe6-N6 2.124(4)	Fe6-N6 2.111(6)

Supplementary Table S4. Bond lengths around the Dy(III) centers within complexes **1 - 3** (in °) in comparison the unsubstituted Fe_6Dy_3 complex
(which crystallized in the monoclinic space group *C2/c*, therefore half the molecule is generated by symmetry operations).

Dy-O	Fe_6Dy_3 (original)	complex **1**	complex **2**	complex **3**
Dy-O_{OMe}	Dy1-O1 2.256(4)	Dy1-O1 2.253(4)	Dy1-O1 2.233(4)	Dy1-O1 2.262(4)
	Dy1-O1' 2.256(4)	Dy1-O9 2.270(4)	Dy1-O9 2.258(4)	Dy1-O9 2.281(4)
	Dy2-O3 2.270(4)	Dy2-O3 2.283(4)	Dy2-O3 2.273(4)	Dy2-O3 2.269(4)
	Dy2-O4 2.270(4)	Dy2-O4 2.275(4)	Dy2-O4 2.270(4)	Dy2-O4 2.265(4)
	Dy2'-O3' 2.270(4)	Dy3-O6 2.258(5)	Dy3-O6 2.248(4)	Dy3-O6 2.257(4)
	Dy2'-O4' 2.270(4)	Dy3-O8 2.251(4)	Dy3-O7 2.244(4)	Dy3-O7 2.253(4)
Dy-$O_{benzoate}$	Dy1-O15 2.309(4)	Dy1-O28 2.311(4)	Dy1-O28 2.309(4)	Dy1-O28 2.332(4)
	Dy1-O15' 2.309(4)	Dy1-O39 2.336(4)	Dy1-O39 2.325(4)	Dy1-O38 2.234(5)
	Dy2-O18 2.334(4)	Dy2-O31 2.318(4)	Dy2-O31 2.309(4)	Dy2-O31 2.353(4)
	Dy2-O19 2.292(4)	Dy2-O32 2.329(4)	Dy2-O32 2.308(4)	Dy2-O32 2.331(4)
	Dy2'-O18' 2.334(4)	Dy3-O35 2.297(5)	Dy3-O35 2.282(4)	Dy3-O35 2.305(4)
	Dy2'-O19' 2.292(4)	Dy3-O36 2.310(5)	Dy3-O36 2.294(4)	Dy3-O36 2.337(4)
Dy-O_{phenol}	Dy1-O6 2.404(4)	Dy1-O10 2.431(4)	Dy1-O10 2.431(4)	Dy1-O10 2.410(5)
	Dy1-O6' 2.404(4)	Dy1-O25 2.388(4)	Dy1-O25 2.392(3)	Dy1-O25 2.366(4)
	Dy2-O9 2.379(4)	Dy2-O13 2.369(5)	Dy2-O14 2.361(4)	Dy2-O13 2.379(4)
	Dy2-O12 2.407(4)	Dy2-O16 2.365(4)	Dy2-O16 2.367(4)	Dy2-O16 2.386(4)
	Dy2'-O9' 2.379(4)	Dy3-O19 2.388(5)	Dy3-O19 2.379(4)	Dy3-O19 2.415(4)
	Dy2'-O12' 2.407(4)	Dy3-O22 2.422(5)	Dy3-O22 2.420(4)	Dy3-O22 2.384(4)
Dy-$O_{OMe(ligand)}$	Dy1-O8 2.526(5)	Dy1-O11 2.516(4)	Dy1-O11 2.509(4)	Dy1-O11 2.477(5)
	Dy1-O8' 2.526(5)	Dy1-O26 2.555(4)	Dy1-O26 2.531(4)	Dy1-O26 2.532(5)
	Dy2-O11 2.544(4)	Dy2-O14 2.499(4)	Dy2-O13 2.511(4)	Dy2-O14 2.466(4)
	Dy2-O14 2.530(5)	Dy2-O18 2.518(5)	Dy2-O17 2.520(4)	Dy2-O17 2.502(5)
	Dy2'-O11' 2.544(4)	Dy3-O20 2.545(5)	Dy3-O20 2.549(5)	Dy3-O20 2.503(4)
	Dy2'-O14' 2.530(5)	Dy3-O23 2.499(5)	Dy3-O23 2.510(5)	Dy3-O23 2.492(4)

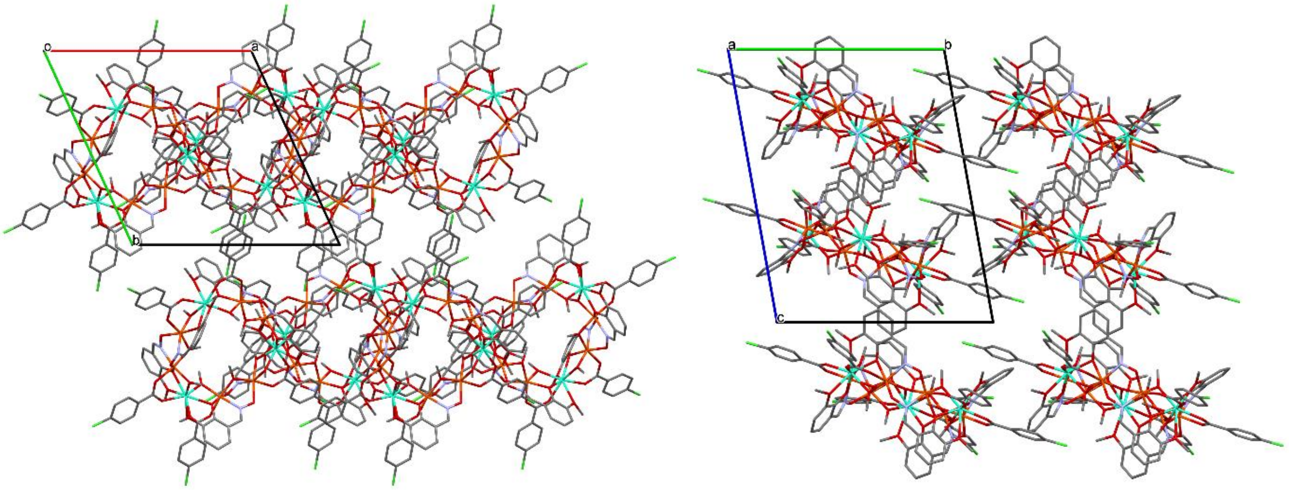

Supplementary Figure 3. Packing arrangement of **2** along *c*-axis (left) and *a*-axis (right). (complex **1** exhibits a similar packing arragement).

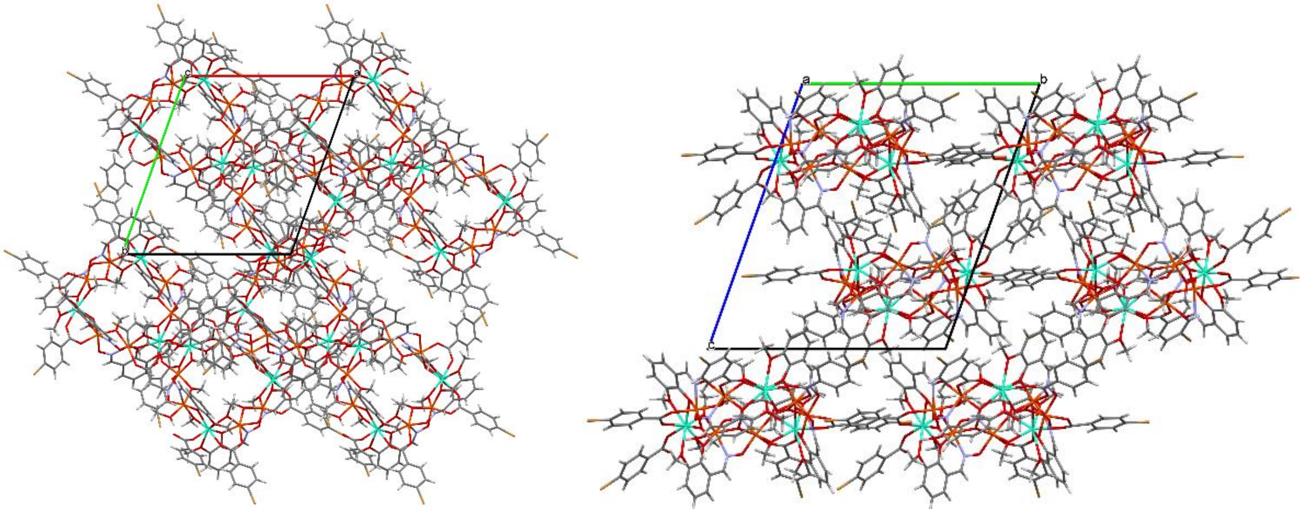

Supplementary Figure 4. Packing arrangement of **3** along *c*-axis (left) and *a*-axis (right).

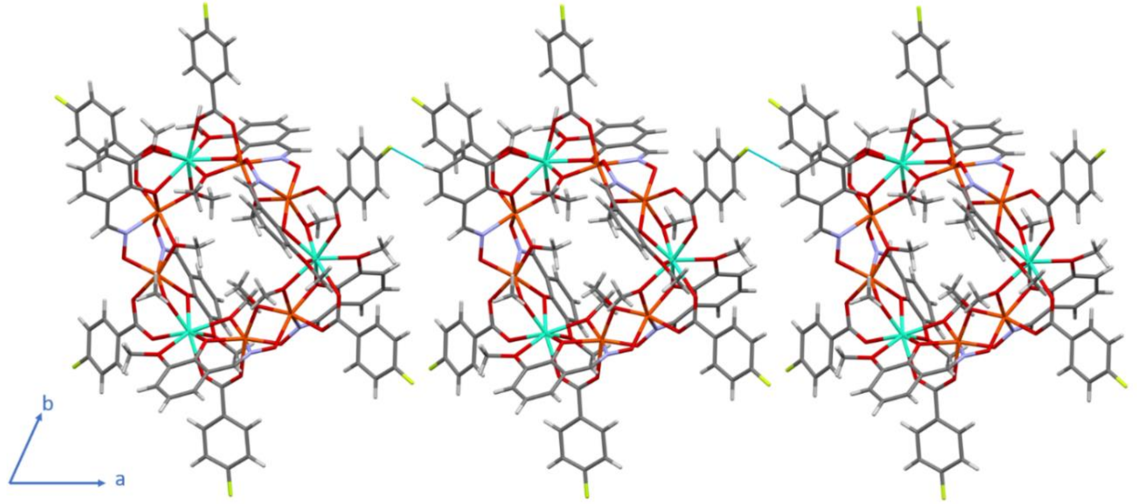

Supplementary Figure 5a. Hydrogen bonds, H-F, between neighbouring molecules within compound **1** shown as turcoise dashed lines, forming chains along a within the ab-plane.

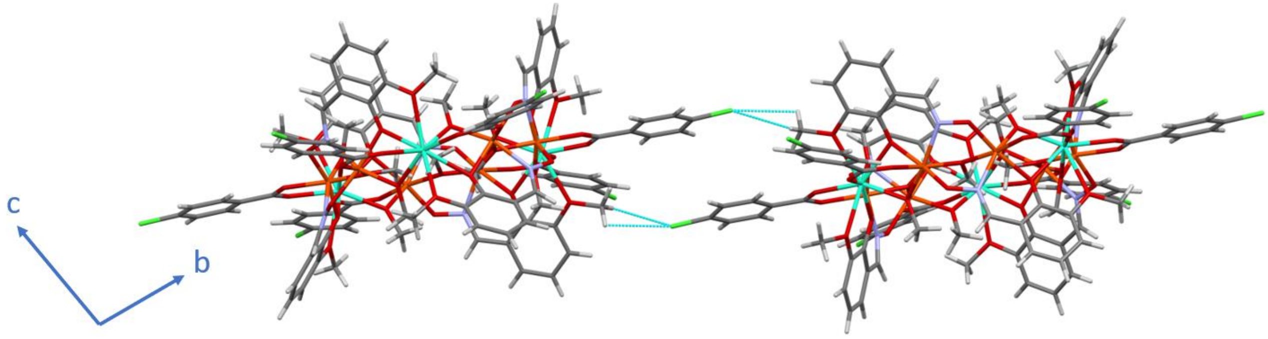

Supplementary Figure 5b. Hydrogen bonds, H-Cl, between two neighbouring molecules within compound **2** shown as turcoise dashed lines.

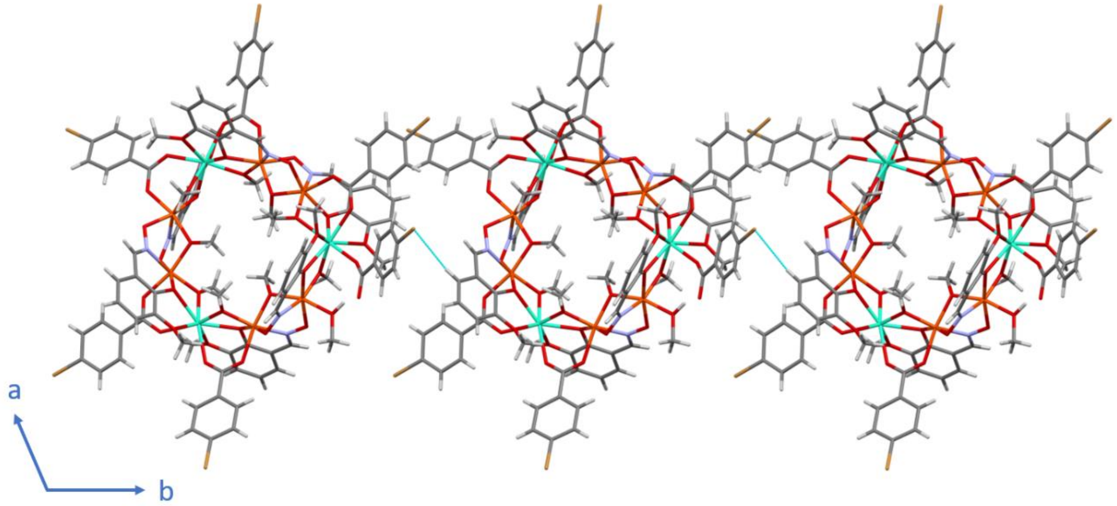

Supplementary Figure 5c. Hydrogen bonds, H-Br, between two neighbouring molecules within compound **3** shown as turcoise dashed lines, forming chains along the *b*-axis within the *ab*-plane.

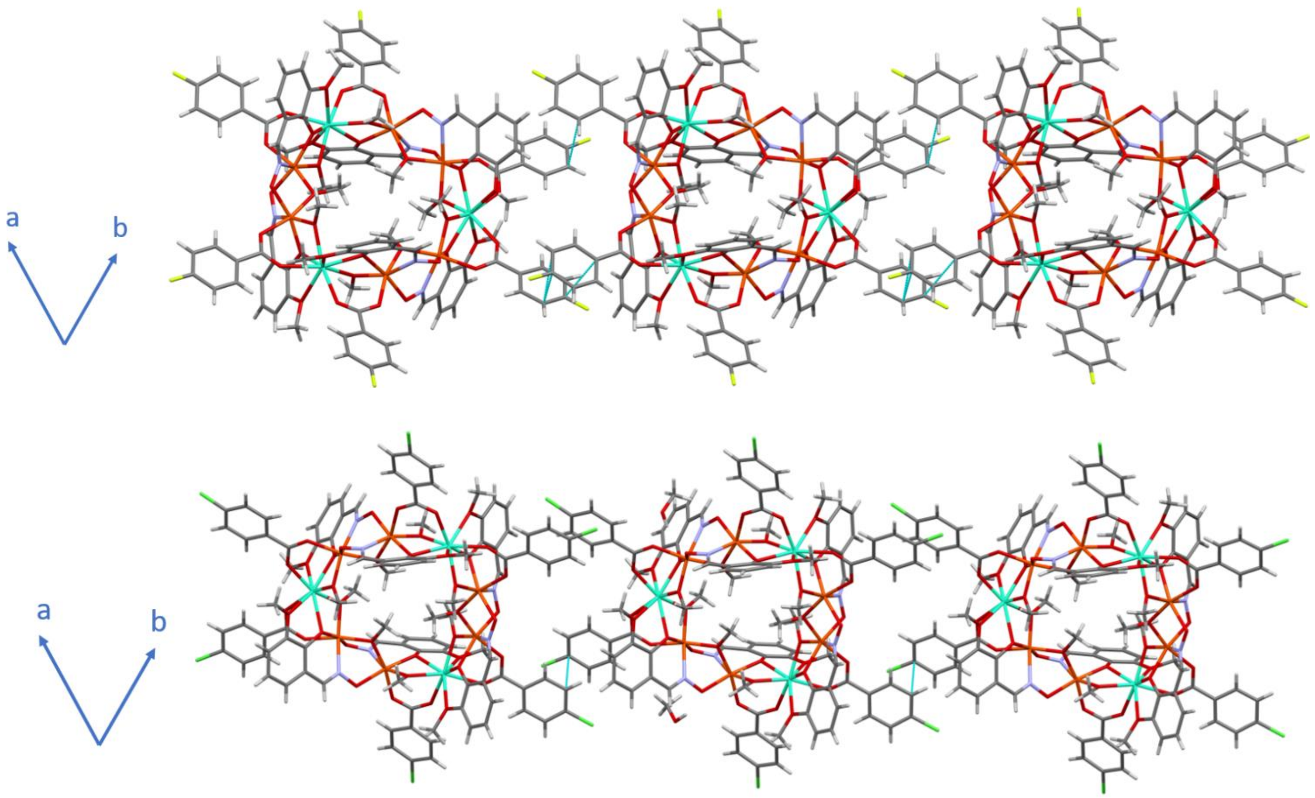

Supplementary Figure 6. Close contacts between neighbouring 4-fluorobenzoate ligands withing complex **1** (top) and neighbouring 4-chlorobenzoate ligands withing complex **2** (bottom) forming sheets of molecules within the *ab*-plane, short contacts shown as dashed turcoise lines.

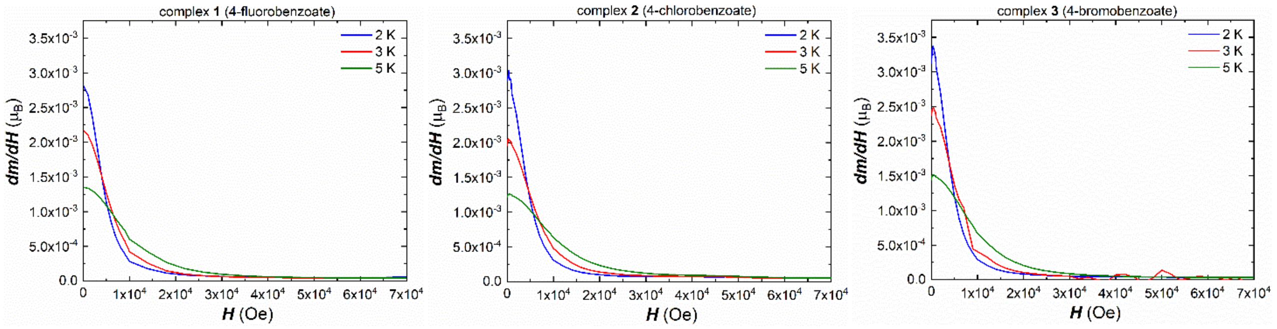

Supplementary Figure 7. The first derivatives of the magnetization with respect to the applied field dM/dH (lines are guides to the eyes) for complexes **1** - **3** at 2 K, 3 K and 5 K.

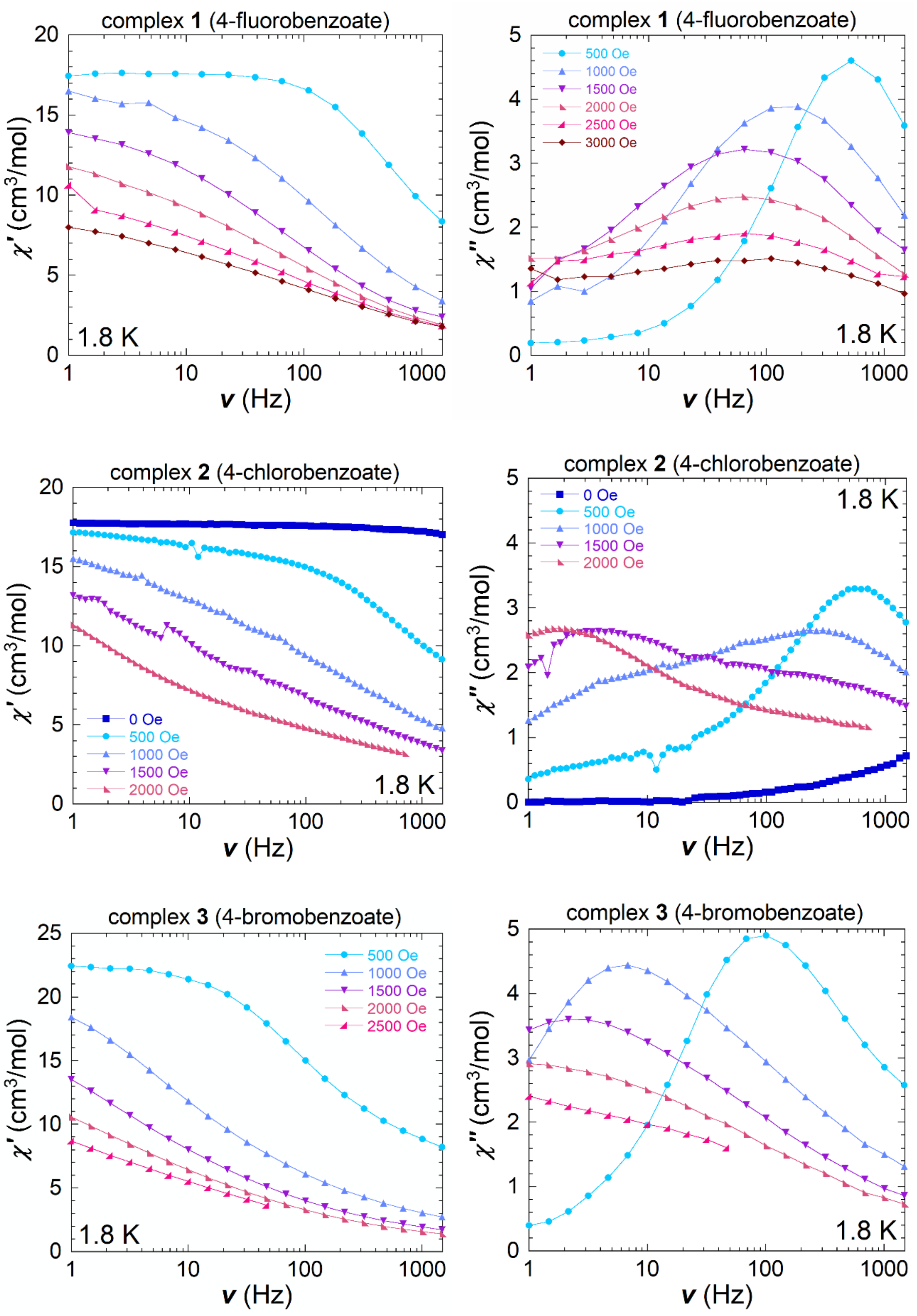

Supplementary Figure 8. In-phase (left) and out-of-phase ac susceptibility (right) at 1.8 K and varying field between 0 Oe and 3000 Oe for complexes **1** - **3**.

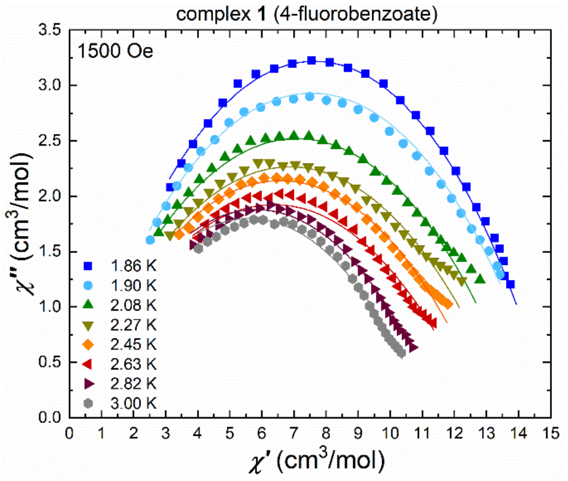

Supplementary Figure 9. Cole-Cole plot between 1.86 and 3.0 K for complex **1** measured at 1500 Oe. (Solid lines are fits of the experimental data using CC-Fit which uses a generalized Debye model).

Supplementary Table S5. Resulting parameters of τ and α obtained with CC-Fit for complex **1** at 1500 Oe.

T(K)	1/T	tau	alpha
1.8652	0.53613	0.0023116	0.47734
1.8986	0.52672	0.0022782	0.52144
2.0830	0.48008	0.0018617	0.56490
2.2663	0.44125	0.0015990	0.59500
2.4497	0.40822	0.0011285	0.59897
2.6327	0.37984	0.00084525	0.62658
2.8161	0.35509	0.00057021	0.60276
2.9996	0.33337	0.00041563	0.60436

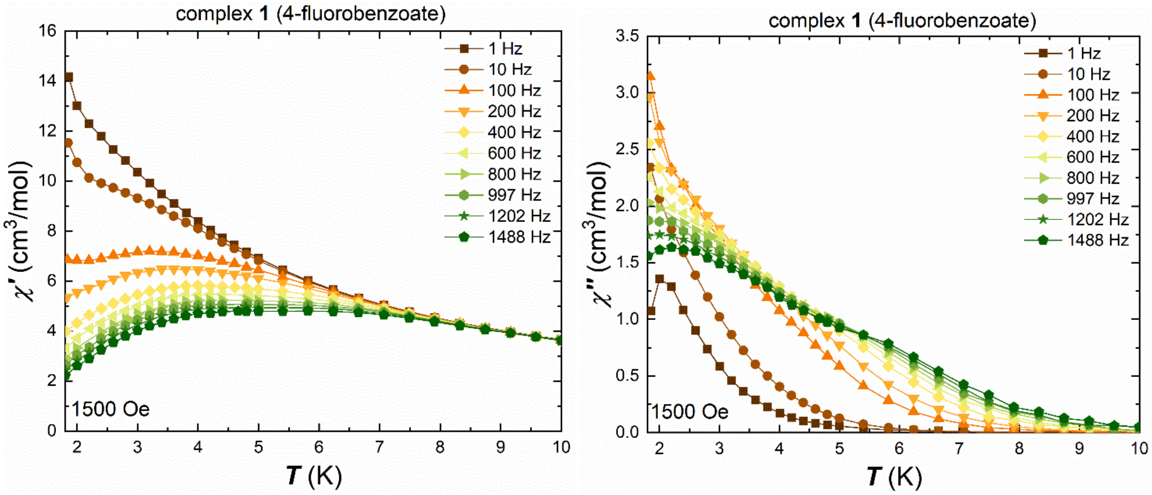

Supplementary Figure 10. Temperature dependent ac susceptibility using different frequencies between 1.0 - 1488.0 Hz, for complex **1** measured under a dc field of 1500 Oe with the in-phase (left) and out-of-phase ac susceptibility (right) in a temperature range between 1.9 - 6.0 K (lines are guides to the eye).

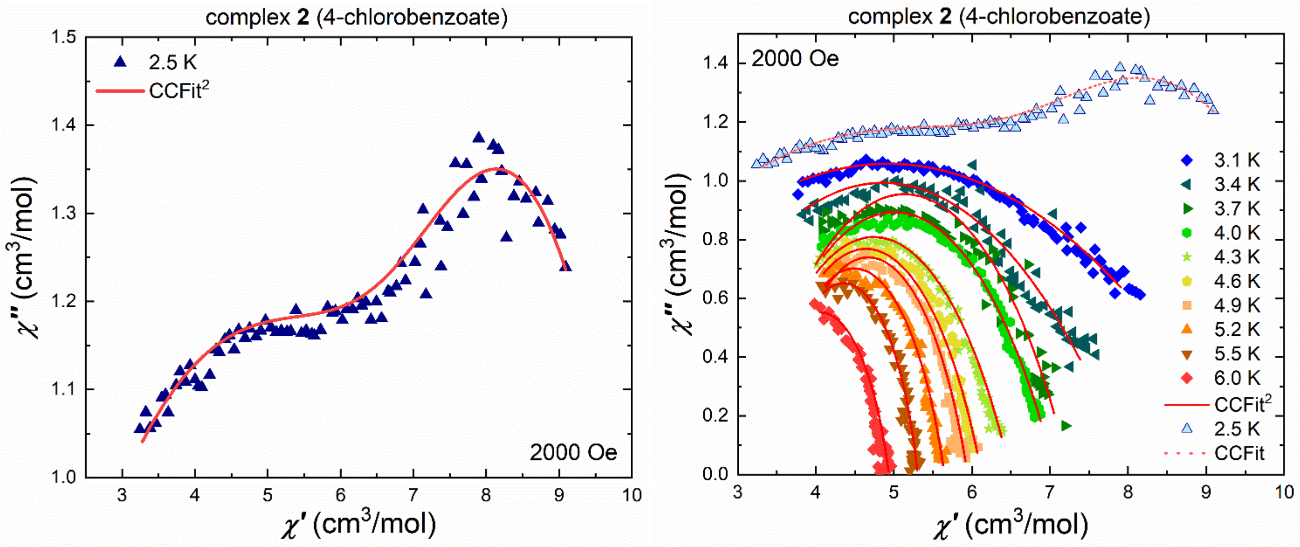

Supplementary Figure 11. Cole-Cole plot for 2.5 K (left) and in the temperature range between 3.1 - 6.0 K (right) for complex **2** measured at 2000 Oe and in-phase and out-of-phase ac susceptibility fit using CCFit^2 in a temperature range between 3.1 - 6.0 K.
(colors of Cole-Cole plot according to Figure 4; solid lines are fits of the experimental data using CC-Fit which uses a generalized Debye model).

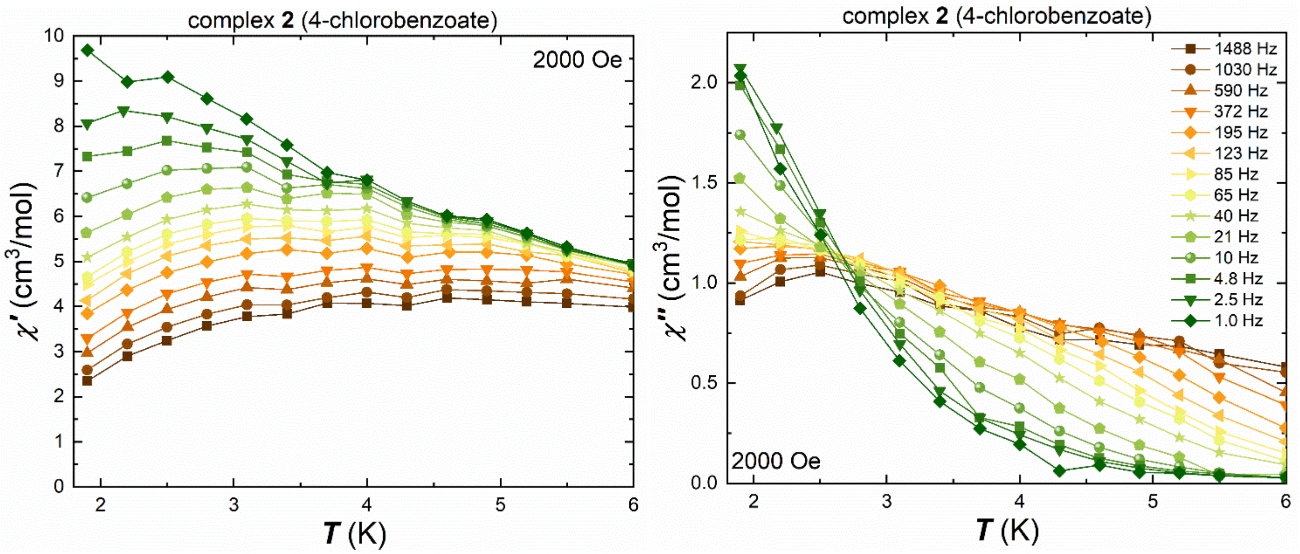

Supplementary Figure 12. Temperature dependent ac susceptibility using different frequencies between 1.0 - 1488.0 Hz, for complex **2** measured under a dc field of 2000 Oe with the in-phase (left) and out-of-phase ac susceptibility (right) in a temperature range between 1.9 - 6.0 K
(lines are guides to the eye).

Supplementary Table S6. Resulting parameters of τ and α obtained with CC-Fit[2] for complex **2** at 2000 Oe**.**

T(K)	1/T	tau	alpha
3.0987	0.32271	0.00055127	0.71564
3.3989	0.29421	0.00049631	0.61431
3.6991	0.27034	0.00069873	0.46123
3.9988	0.25008	0.00050123	0.47955
4.2994	0.23259	0.00041443	0.44926
4.6016	0.21732	0.00029175	0.38976
4.8988	0.20413	0.00029560	0.32892
5.1986	0.19236	0.00021992	0.30461
5.4987	0.18186	0.00017513	0.23271
5.9987	0.16670	0.00010828	0.28191

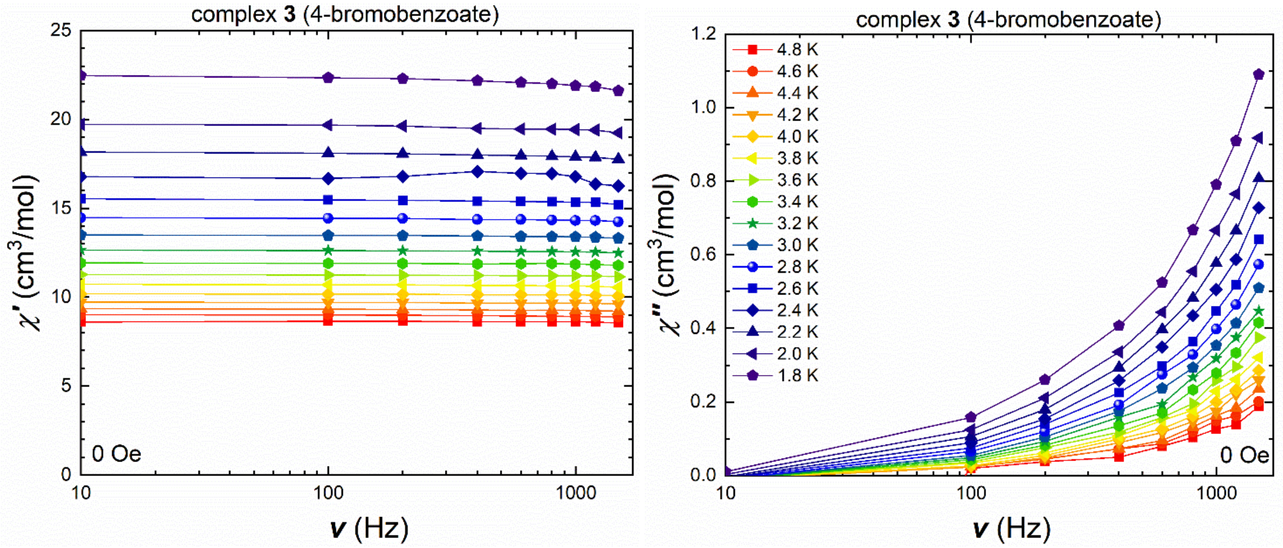

Supplementary Figure 13. Frequency-dependent in-phase (left) and out-of-phase susceptibility (right) without applied field of complex **3** in a temperature range between 1.8 - 4.8 K (lines are guides to the eye).

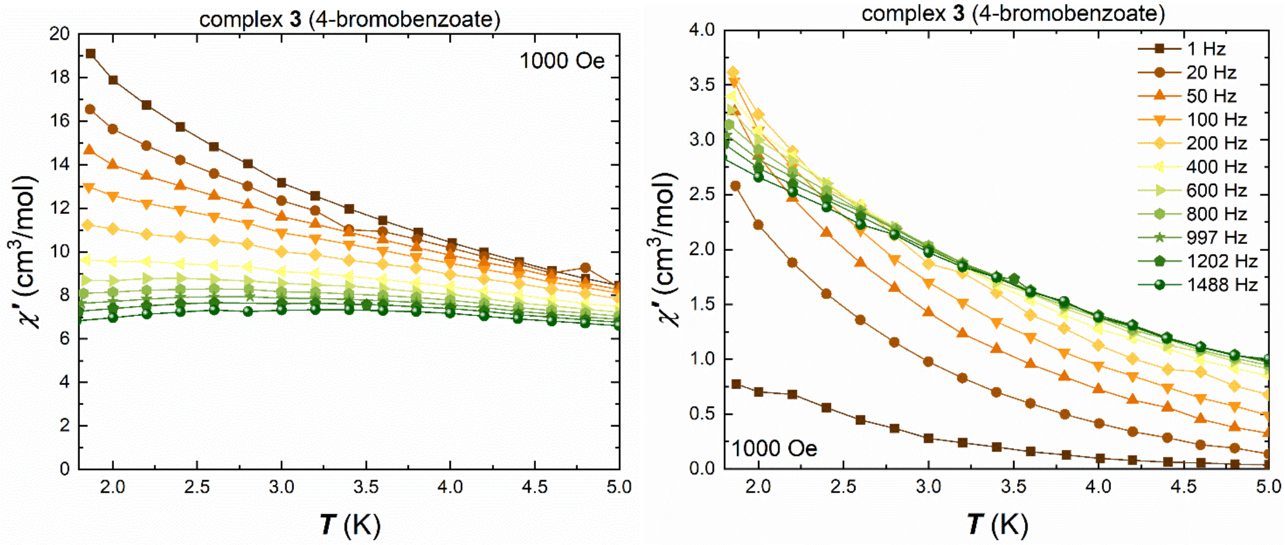

Supplementary Figure 14. Temperature dependent ac susceptibility using different frequencies between 1.0 - 1488.0 Hz, for complex **3** measured under a dc field of 1000 Oe with the in-phase (left) and out-of-phase ac susceptibility (right) in a temperature range between 1.9 - 5.0 K
(lines are guides to the eye).

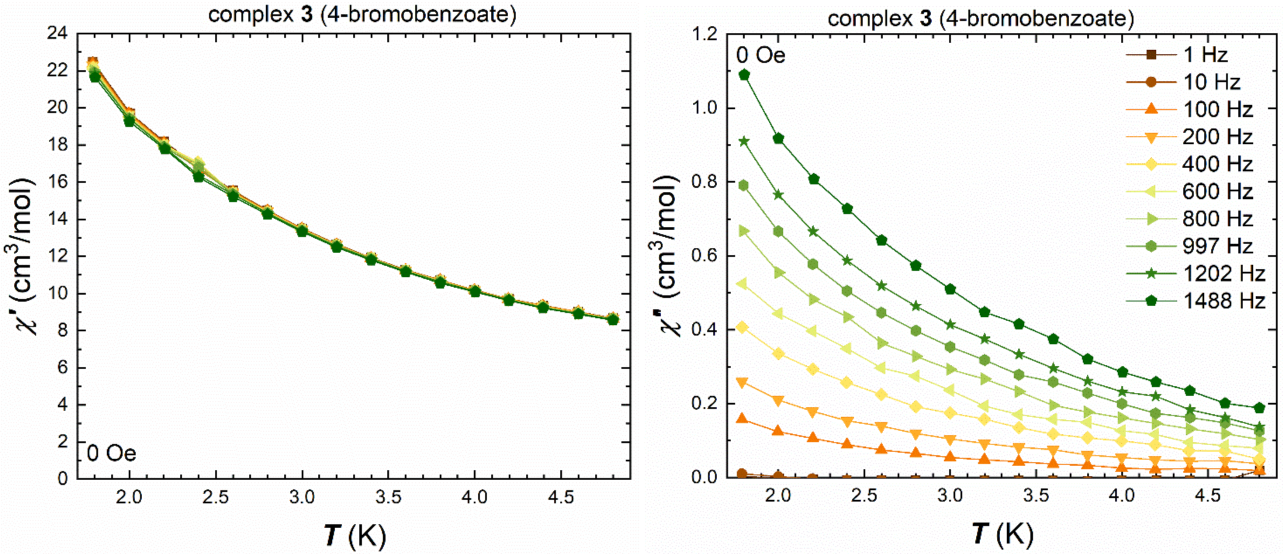

Supplementary Figure 15. Temperature dependent ac susceptibility using different frequencies between 1.0 - 1488.0 Hz, for complex **3** measured without applied dc field with the in-phase (left) and out-of-phase ac susceptibility (right) in a temperature range between 1.9 - 4.8 K
(lines are guides to the eye).

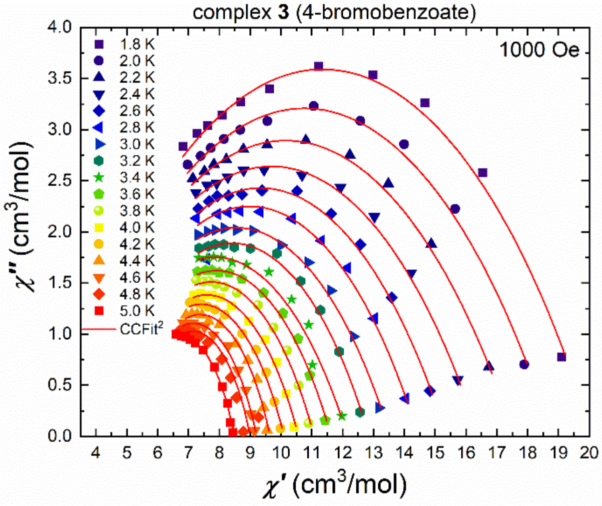

Supplementary Figure 16. Cole-Cole plot between 1.8 and 5.0 K for complex **3** measured at 1000 Oe using CCFit[2] over the whole measured temperature range
(colors of Cole-Cole plot according to Figure 5); solid lines are fits of the experimental data using CC-Fit which uses a generalized Debye model).

Supplementary Table S7. Resulting parameters of τ and α obtained with CC-Fit[2] for complex **3** at 1000 Oe.

T(K)	1/T	tau	alpha
1.83692	0.54439	0.00079385	0.50408
2.00024	0.49994	0.00067839	0.51634
2.20017	0.45451	0.00053637	0.52632
2.40001	0.41667	0.00043840	0.52336
2.59991	0.38463	0.00037632	0.51232
2.80173	0.35692	0.00032020	0.50379
2.99954	0.33338	0.00026241	0.50608
3.19962	0.31254	0.00024602	0.48801
3.39973	0.29414	0.00016690	0.50828
3.59961	0.27781	0.00018596	0.47593
3.79879	0.26324	0.00017501	0.45672
3.99998	0.25	0.00016837	0.44388
4.19924	0.23814	0.00016004	0.4284
4.40392	0.22707	0.00018285	0.38404
4.59909	0.21743	0.00016468	0.37201
4.79895	0.20838	0.00018961	0.35666
4.9989	0.20004	0.00013255	0.34745

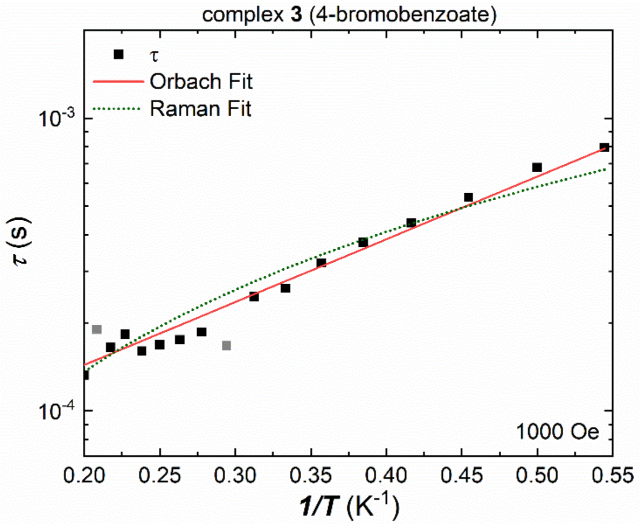

Supplementary Figure 17. Extracted τ values plotted against 1/T (black squares) and simultaneous Orbach-QTM fit (red line) and for Raman-QTM fit (green dashed line) for complexes **3**, leading to U_{eff} = 4.94 K and τ_0 = $5.24 \cdot 10^{-5}$ s for the Orbach process (red), and a Raman contribution (green) with $1/\tau \propto T^n$ with n = 1.59 and C = $5.7 x 10^2$ $s^{-1}K^{-n}$ (gray squares omitted in the fit).

The fit of the extracted τ values as a Raman relaxation process is not suitable for this compound, since first of all the exponent n does not correspond a good value (n can vary depending on the exact energies of the ground doublets but only values are reasonable for n ≥ 4) and secondly the shape of the extracted Raman process does not fit the linear behavior of the τ values, which is indicative of an exponential behavior; therefore a Raman relaxation fit is redundant, but shown for completeness.

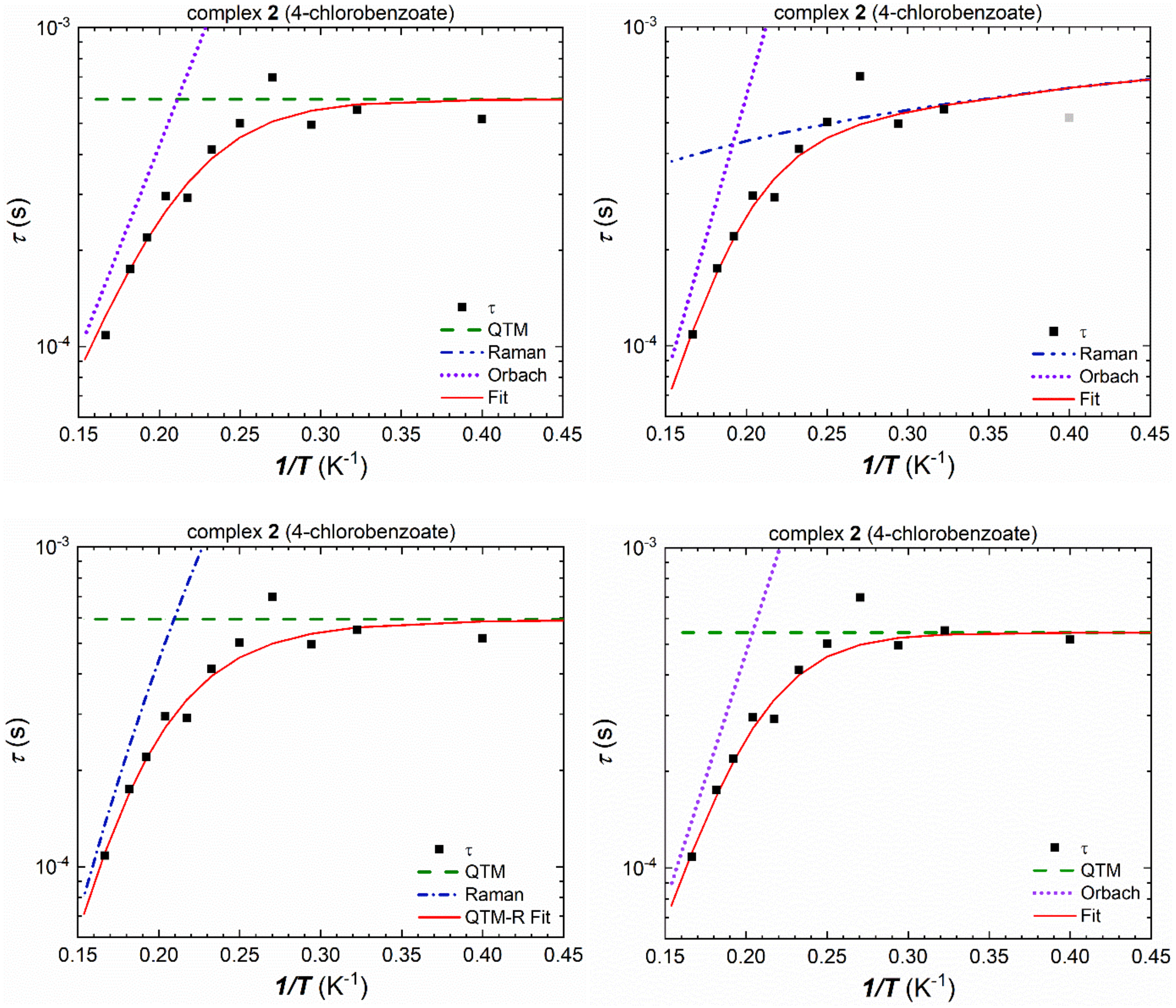

Supplementary Figure 18. Extracted τ values plotted against 1/T (black squares) for complex **1**; simultaneous Orbach-Raman-QTM fit (top left), simultaneous Orbach-Raman fit (top right), simultaneous Raman-QTM fit (bottom left) and simultaneous Orbach-QTM fit (bottom right) with QTM (green dashed lines), Raman contribution (blue dashed lines), Orbach contribution (purple dotted lines) leading to the best fit (red solid line).

Extracted parameters:

1.) simultaneous Orbach-Raman-QTM fit (top left): Orbach: U_{eff} = 29.8 K, τ_0 = $1.09 \cdot 10^{-6}$ s; Raman contribution with $1/\tau \propto T^n$ with n = 197.3 and C = $3.99 \cdot 10^{-151} \approx 0$ $s^{-1}K^{-n}$ (these parameters are indicative that the fit does not require any Raman process); and quantum tunneling parameter τ_{QTM} = $10^{-3.22}$ s = $5.95 \cdot 10^{-4}$ s. This simultaneous fit shows that in the measured temperature range the relaxation pathway can be fitted using only an Orbach and a QTM process, while the Raman part is redundant.

2.) simultaneous Orbach-Raman fit (top right): By leaving out the QTM part at low temperatures, in attempt to improve the fit, this led to the following parameters: Orbach: U_{eff} = 41.0 K; τ_0 = $1.65 \cdot 10^{-7}$ s; and a Raman contribution with $1/\tau \propto T^n$ with n = 0.55 and C = $9.34 \cdot 10^{2}$ $s^{-1}K^{-n}$. This shows again, that a fit using a Raman contribution is not suitable for this compound, since this time the exponent n does not correspond a good value (n can vary depending on the exact energies of the ground doublets but only values are reasonable for n ≥ 4); therefore this fit is redundant, but shown for completeness.

3.) simultaneous Raman-QTM fit (bottom left): It is vital to consider all possible combinations of thermally induced relaxation processes, such as the last combination to include a Raman contribution with a QTM process, leading to the following parameters: Raman contribution with $1/\tau \propto T^n$ with n = 6.47 and C = $6.74 \cdot 10^{-2}$ $s^{-1}K^{-n}$; and quantum tunneling parameter τ_{QTM} = $10^{-3.22}$ s = $5.93 \cdot 10^{-4}$ s. This fit clearly shows that the QTM parameter is essentially the same as in the first case and has to be included in the fit, while the Raman process physically yields reasonable values, which are similar to other Dy(III) containing systems.

4.) simultaneous Orbach-QTM fit (bottom right): Orbach: U_{eff} = 36.1 K, τ_0 = $3.44 \cdot 10^{-7}$ s; and quantum tunneling parameter τ_{QTM} = $10^{-3.26}$ s = $5.43 \cdot 10^{-4}$ s.

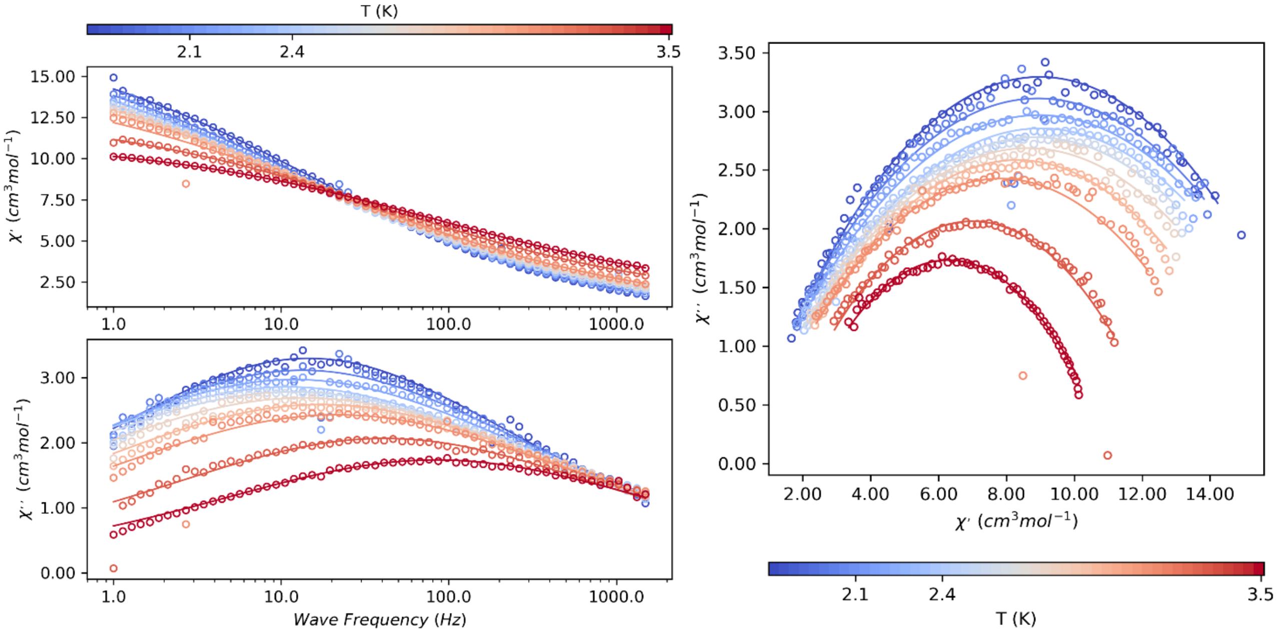

Supplementary Figure 19. Frequency-dependent in-phase and out-of phase susceptibility under an external dc field of 2000 Oe (left) for the unsubstituted Fe_6Dy_3 complex in a temperature range between 1.8 - 3.5 K; Cole-Cole plots (right) between 1.8 and 3.5 K using CCFit[2] over the whole measured temperature range (right)
(solid lines are fits of the experimental data fitted with a generalized Debye model).

Supplementary Table S8. Resulting parameters of τ and α obtained with CC-Fit for the unsubstituted Fe_6Dy_3 compound.

T(K)	1/T	tau	alpha
1.8	0.55556	0.01092	0.54257
1.9	0.52632	0.01187	0.57349
2.0	0.5	0.0133	0.59332
2.1	0.47619	0.01451	0.60047
2.2	0.45455	0.01362	0.60701
2.3	0.43478	0.01164	0.60805
2.4	0.41667	0.00921	0.59586
2.5	0.4	0.0074	0.59663
3.0	0.33333	0.00384	0.5696
3.5	0.28571	0.00189	0.56669

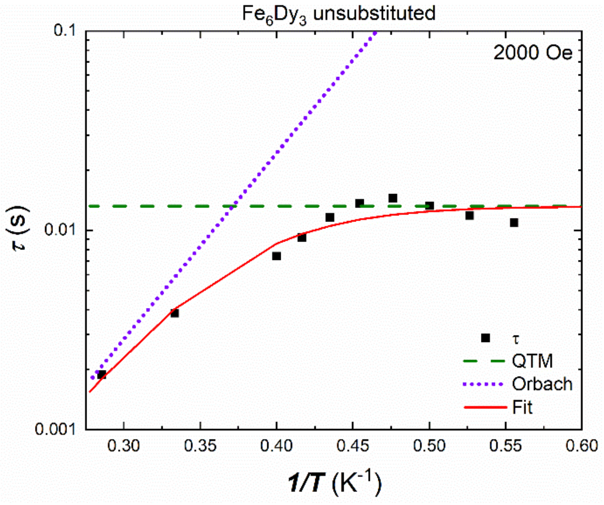

Supplementary Figure 20. Extracted τ values plotted against 1/T (black squares) for the unsubstituted Fe_6Dy_3 complex and the simultaneous Orbach-QTM fit
with QTM (green dashed lines) and the Orbach contribution (purple dotted lines) leading to the best fit (red solid line).

→ simultaneous Orbach-QTM fit: Orbach: U_{eff} = 21.5 K, $\tau_0 = 4.49 \cdot 10^{-6}$ s; and quantum tunneling parameter $\tau_{QTM} = 10^{-1.87}$ s = $1.32 \cdot 10^{-2}$ s.

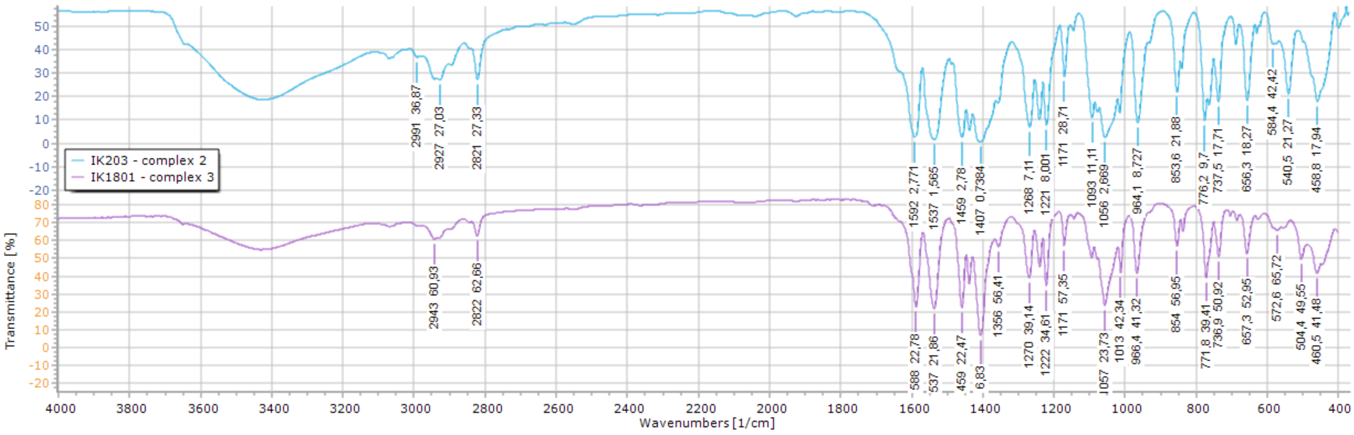

Supplementary Figure S21. Comparison of Infrared (IR) spectra of compounds **2** (4-chloro, blue) and **3** (4-bromo, purple).

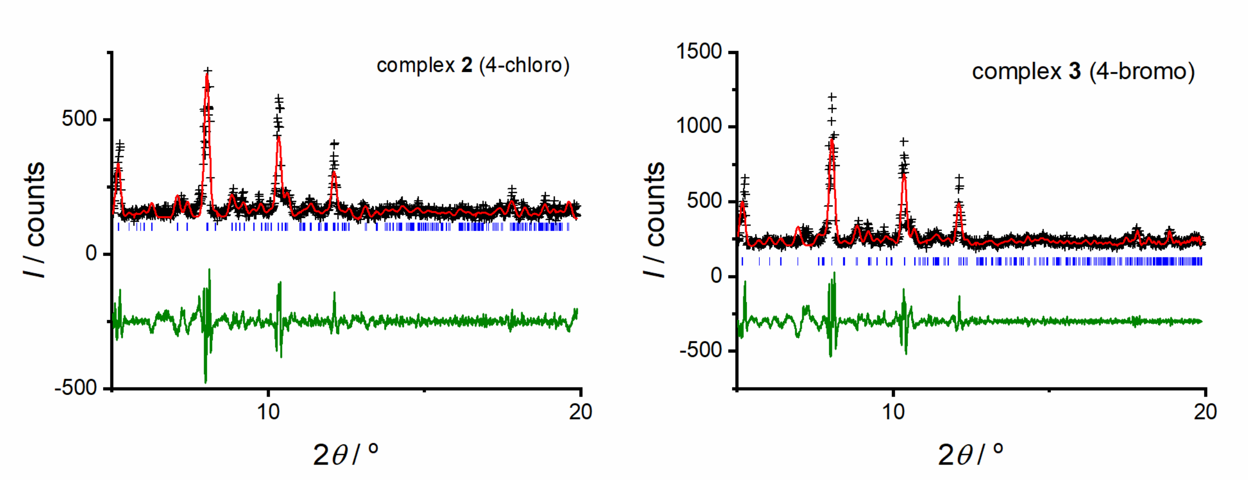

Supplementary Figure S22. PXRD pattern (black), fit (red), predicted peak position (blue) and difference between predicted and calculated pattern (green) for dried polycrystalline complex **2** (left) and complex **3** (right), leading to Rp = 8.93 (complex **2**) and Rp = 8.14 (complex **3**).

CELL PARAMETERS IK203 = complex **2**

18.252724	-0.000170	0.012737
19.401009	0.000038	0.009954
23.096556	-0.000044	0.010139
74.288429	0.000290	0.030400
74.183418	0.000053	0.050734
62.366730	-0.000290	0.050895

CELL PARAMETERS IK1801 = complex **3**

16.643385	-0.000008	0.005427
20.277775	-0.000023	0.006373
22.638458	-0.000006	0.004694
109.283661	0.000008	0.018994
94.047485	0.000137	0.036477
108.680931	0.000015	0.026743
